# Lactobionic Acid as a Biocompatibility Switch: Physicochemical and Biological Characterization of Copper(II)–Primaquine Complexes

**DOI:** 10.1155/bca/9997326

**Published:** 2026-09-09

**Authors:** Alessia Distefano, Laura Cifalinò, Alessia Sambugaro, Erik Murador, Alessandra Folda, Giuseppe Grasso, Chiara Nardon, Maria Pia Rigobello, Valeria Scalcon, Valentina Oliveri

**Affiliations:** ^1^ Dipartimento di Scienze Chimiche, Università Degli Studi di Catania, v.le A. Doria 6, Catania 95125, Italy, unict.it; ^2^ Dipartimento di Biotecnologie, Università Degli Studi di Verona, Strada le Grazie 15, Verona 37134, Italy, univr.it; ^3^ Dipartimento di Scienze Biomediche, Università Degli Studi di Padova, via Ugo Bassi 58/b, Padova 35131, Italy, unipd.it

## Abstract

Metal dyshomeostasis and oxidative stress are implicated in the progression of cancer, and neurodegenerative and peripheral aggregation‐related disorders. In this study, we investigated primaquine (PQ), a clinically used antimalarial drug, and PQ–lactobionic acid conjugate (LAPQ), a newly synthesized and characterized derivative obtained through conjugation with lactobionic acid, designed to improve physicochemical and biological properties. Both compounds were evaluated for their copper‐coordination ability, antioxidant properties, interaction with amyloid‐β (Aβ), and capacity to modulate reactive oxygen species (ROS)‐induced cellular damage. As a complementary biological line of investigation, their in vitro antiproliferative activity in the presence and absence of copper was also evaluated. The compounds directly interact with Aβ as demonstrated by surface plasmon resonance (SPR) studies. Biological studies revealed marked differences between the two molecules. PQ displayed intrinsic cytotoxicity, whereas LAPQ exhibited enhanced aqueous solubility and substantially reduced antiproliferative activity under the tested conditions, highlighting the impact of sugar conjugation. In cellular oxidative stress models, LAPQ showed a protective effect, preserving cell viability under ROS‐generating conditions. In summary, by interacting with Aβ and exhibiting antioxidant activity, two properties relevant to several neurodegenerative and peripheral aggregation‐related disorders, our novel compound LAPQ may provide a potential starting point for the development of therapies targeting Aβ–associated disorders.

## 1. Introduction

Primaquine (PQ) is an 8‐aminoquinoline (8‐AQ) antimalarial drug that has been used clinically for approximately 70 years. It is effective against chloroquine‐resistant *Plasmodium falciparum* strains and plays a critical role in preventing disease relapses caused by *Plasmodium vivax* and *Plasmodium ovale* by eliminating dormant parasites in the liver (hypnozoites) and blocking transmission through gametocytes. The World Health Organization (WHO) continues to recommend PQ as part of transmission‐blocking strategies in regions with low malaria transmission intensity, recognizing its irreplaceable role in malaria elimination efforts [1]. However, despite its importance as part of radical treatment regimens, the clinical application of PQ remains significantly restricted due to serious safety concerns [2], particularly the risk of hemolytic anemia in individuals with glucose‐6‐phosphate dehydrogenase (G6PD) deficiency, an X‐linked disorder with over 200 genetic variants and differing susceptibility to oxidative stress. Additional side effects include methemoglobinemia, leukopenia, abdominal cramps, and epigastric distress. Careful screening and monitoring are vital when prescribing this drug [2, 3]. These documented adverse effects have motivated researchers to explore PQ structural derivatization as a pathway toward safer and more therapeutically versatile compounds [4].

Moreover, the pharmacophore of the 8‐AQ scaffold possesses substantial intrinsic biological activity that merits exploration for applications beyond malaria treatment [5–7]. Various antimalarial drugs show direct or adjuvant anticancer effects [8, 9]. Research over the past 2 decades has demonstrated that PQ exhibits significant anticancer activity through multiple mechanisms, including DNA damage and the production of reactive oxygen species (ROS) [10]. Modifying the structure of PQ, particularly at the terminal amino group, resulted in derivatives that selectively inhibit cancer cell growth while exhibiting less toxicity to normal cells [11]. Furthermore, other 8‐AQ derivatives and their complexes have been shown to possess neuroprotective properties through modulation of sirtuins and oxidative stress pathways, demonstrating the remarkable versatility of this scaffold for therapeutic applications [12]. Recognizing the potential of PQ as a privileged scaffold for developing anticancer and protective agents, researchers have explored rational chemical derivatization strategies aimed at both reducing toxicity to normal tissues and enhancing/adding therapeutic effects [13].

Lactobionic acid (LA), a polyhydroxy acid composed of galactose and gluconic acid, is known for its remarkable biological properties, including antimicrobial activity against *Staphylococcus aureus* [14, 15], antioxidant capacity, prebiotic effects supporting beneficial bacteria [16], and excellent biocompatibility [17]. Its incorporation into drug structures has demonstrated significant potential for several reasons: first, LA contains a galactose moiety that is recognized by asialoglycoprotein receptors (ASGPRs) on hepatocyte surfaces [18, 19]; second, LA conjugation dramatically improves water solubility of macromolecules, as demonstrated with chitosan‐based systems [20]; third, conjugation provides controlled release of the therapeutic cargo while preserving the biological activity of the acid [21]; fourth, LA enables the design of multifunctional biomaterials that exhibit synergistic therapeutic effects [22]; and finally, LA has Food and Drug Administration (FDA)–approval status, which facilitates regulatory pathways for developing conjugated therapeutics [15].

**FIGURE 1:**
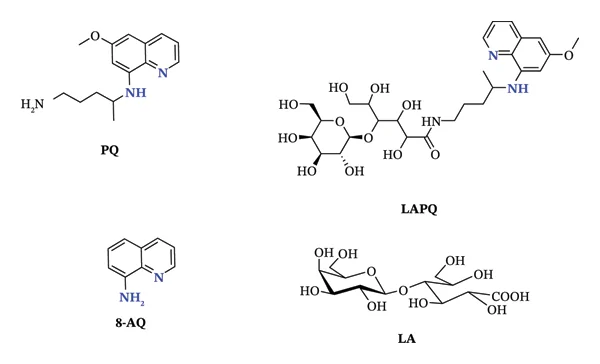
Chemical structures of primaquine (PQ), the corresponding lactobionic acid (LA) derivative (LAPQ), 8‐aminoquinoline (8‐AQ), and lactobionic acid (LA).

In this study, we report the synthesis and full characterization of a PQ–lactobionic acid conjugate (LAPQ, Figure 1). Selected biological effects of LAPQ were evaluated in comparison with the parent compound PQ, and the copper(II) complexation properties of both were investigated.

Copper(II) plays a significant role in many biological processes and has been widely explored in the design and development of metallodrugs due to their ability to modulate ROS and interfere with cellular redox homeostasis. The study of copper–PQ complexes in cell lines builds upon a broader literature demonstrating the efficacy of diverse copper coordination compounds as anticancer agents and the hypothesized behavior of 8‐AQ as a copper ionophore [23]. In this latter case, as well as in similar compounds based on the 8‐hydroxyquinoline (8‐HQ) scaffold [24, 25], the presence of copper(II) markedly enhances the antiproliferative activity of the organic ligand. The mechanism is most likely related to an ionophoric behavior of the complex, which facilitates intracellular copper accumulation. The resulting copper overload can trigger cuproptosis, a mechanism of cell death associated with mitochondrial dysfunction. In this context, copper complexes represent a particularly promising strategy for the development of a new generation of anticancer agents that exploit copper‐dependent vulnerabilities in tumor cells. Notably, the complexation of PQ with copper has not been systematically investigated so far. Aside from a few isolated reports dating back to the 1980s [26, 27], its Cu(II) coordination chemistry remains largely unexplored, and its relationship with antiproliferative activity has not been addressed, highlighting a significant gap that warrants further investigation. The coordination behavior of PQ and LAPQ was investigated using different techniques, including UV–vis spectrophotometry, circular dichroism (CD), and electrospray ionization–mass spectrometry (ESI–MS) analysis. As for the biological activity, the interaction of these molecules with amyloid‐β (Aβ), a biologically relevant peptide involved in several neurodegenerative and peripheral aggregation‐related disorders (e.g., glaucoma, age‐related macular degeneration, inclusion body myositis, Alzheimer’s disease (AD), and cerebral amyloid angiopathy), was investigated through surface plasmon resonance (SPR).

Furthermore, the antioxidant activity of the ligands with or without copper(II) was evaluated in vitro through free radical scavenging assays (Trolox equivalent antioxidant capacity [TEAC] determination and ascorbate (AA) oxidation prevention) and in a cellular model evaluating the cytoprotective effect against an oxidative stimulus. In particular, the protective effect of PQ and LAPQ against oxidative damage induced by tert‐butyl hydroperoxide (TbOOH) was evaluated in HepG2 cells. As a complementary biological investigation aimed at exploring the pharmacological versatility of the PQ scaffold, the potential antiproliferative effect of the free ligands and their copper(II) complexes on selected cancer cell lines (HepG2, A549, HCT116, and PC3) was evaluated. This analysis allowed us to assess the influence of both LA conjugation and Cu(II) coordination on the cellular activity of the ligands, highlighting the reduced cytotoxicity of LAPQ compared with the parent compound PQ.

## 2. Experimental

### 2.1. Chemicals and Materials

Commercially available reagents/solvents, including 8‐(4‐amino‐1‐methylbutylamino)‐6‐methoxyquinoline diphosphate salt (PQ), 4‐O‐β‐D‐galactopyranosyl‐D‐gluconic acid (LA), 3‐(N‐morpholino)propanesulfonic acid (MOPS), sterile dimethyl sulfoxide (DMSO), 3‐(4,5‐dimethylthiazol‐2‐yl)‐2,5‐diphenyltetrazolium bromide (MTT), reduced glutathione (GSH), fetal bovine serum (FBS), N,N′‐dicyclohexylcarbodiimide (DCC), N‐hydroxysuccinimide (NHS), CuCl_2_ dihydrate, and *n*‐octanol, were purchased from Merck. Dulbecco’s modified Eagle’s medium (DMEM) w/GlutaMAX‐I (pyruvate 1 mM) cell growth medium, an antibiotic mixture of penicillin and streptomycin (5000 U mL^−1^), minimum essential medium (MEM) nonessential amino acid solution (100×), and phosphate‐buffered saline (PBS) were purchased from Thermo Fisher Life Technologies. MEM was purchased from Euroclone. All chemicals were of high grade and used as purchased without further purification. For all experiments conducted in the presence of copper, except for the cell‐based experiments, stock solutions of Cu^2+^ were prepared by dissolving the corresponding perchlorate salt in water. The resulting solutions were then titrated with standardized EDTA.

### 2.2. Synthesis of 2,3,5,6‐tetrahydroxy‐N‐(4‐((6‐methoxyquinolin‐8‐yl)amino)pentyl)‐4‐(((2S,3R,4S,5R,6R)‐3,4,5‐trihydroxy‐6‐(hydroxymethyl)tetrahydro‐2H‐pyran‐2‐yl)oxy)hexanamide

DCC (46 mg, 0.22 mmol) and NHS (26 mg, 0.22 mmol) were added to a solution of LA (80 mg, 0.22 mmol) in dry DMF (6 mL). The reaction was stirred for one hour, and PQ (96 mg, 0.53 mmol) was then added to the mixture, which was stirred for 24 h at room temperature. The solvent was removed in vacuo, and the crude residue was solubilized in water, then filtered and purified first by flash chromatography on a C‐8 column and then on a Sephadex‐CM 25 cation exchange column (HCO_3_^–^ form). The purity was > 95% as confirmed by HPLC analysis.

 Yield: 68%; TLC: R_f_ = 0.58 (AcOEt/PrOH/H_2_O/NH_3_ 4:3:2:1);
 UV–vis (MOPS, pH 7.4): *λ* (*ε*) = 260 (14,900), 353 (2370);
 
^1^H NMR (600 MHz, D_2_O, apparent pH = 6.98): *δ* = 8.46 (m, 1H, H‐2 of PQ), 8.06 (d, *J* = 8.8 Hz, 1H, H‐4 of PQ), 7.38 (dd, *J* = 8.6, 4.5 Hz, 1H, H‐3 of PQ), 6.57 (m, 1H, H‐5 of PQ), 6.44 (s, 1H, H‐7 of PQ), 4.31 (dd, *J* = 14.2, 7.7 Hz, 1H, H‐1′ of LA), 4.23 (dd, *J* = 12.8, 3.6 Hz, 1H, H‐2 of LA), 4.02 (m, 1H, H‐3 of LA), 3.83 – 3.49 (m, 11H, H12 and Hs‐18 of PQ, H‐4, H‐5, H‐6A, H‐6B, H‐4′, H‐5′, H‐6′A, H‐6′B of LA), 3.45–3.36 (m, 2H, H‐2′ and H‐5′ of LA), 3.33 (dd, *J* = 10.1, 3.5 Hz, 1H, H‐3′ of LA), 3.21 (m, 1H, H‐15A of PQ), 3.12 (m, 1H, H‐15B of PQ), 1.56 (m, 4H, Hs‐13 and Hs‐14 of PQ), 1.18 (d, *J* = 6.7 Hz, 3H, Hs‐16 of PQ);
 
^13^C NMR (151 MHz, D_2_O) *δ* = 178.7 (C1 of LA), 160.7 (C6 of PQ), 147.9 (C2 of PQ), 146.7 (C8 of PQ), 138.6 (C4 of PQ), 136.6 (C9 of PQ), 132.4 (C10 of PQ), 124.7 (C3 of PQ), 105.8 (C1′ of LA), 101.3 (C7 of PQ), 96.5 (C5 of PQ), 82.9 (C4 of LA), 77.6 (C5′ of LA), 74.6 (C2 of LA), 73.1(C5 of LA), 72.4 (C3 of LA), 70.7 (C4′ of LA), 64.1 (C6 of LA), 63.2 (C6′ of LA), 57.6 (C18 of PQ), 50.2 (C12 of PQ), 41.1 (C15 of PQ), 35.1 (C13 of PQ), 27.1 (C14 of PQ), 21.50 ppm (C16 of PQ).
 MS (ESI+): m/z calcd for [C_27_H_42_N_3_O_12_]^+^: 600.27; found: 600.22 [M+H]^+^.

### 2.3. In Silico Absorption, Distribution, Metabolism, Excretion, and Toxicity (ADMET) and Drug‐Likeness Analysis

The newly synthesized compound LAPQ was subjected to in silico ADMET and drug‐likeness analysis to obtain a preliminary assessment of its pharmacokinetic and toxicological properties. The molecular structure of LAPQ was generated using ChemDraw and used as input for the SwissADME and ADMETlab 3.0 web platforms [28, 29]. Predictions included physicochemical descriptors, drug‐likeness parameters, absorption, distribution, metabolism, excretion, and toxicity properties. The use of two independent prediction tools allowed a comparative evaluation of the calculated parameters and increased the reliability of the computational assessment. Given the intrinsic limitations of in silico models, including applicability‐domain restrictions, training‐set bias, and inter‐platform variability, the obtained results were considered preliminary indicators of the pharmacokinetic behavior of LAPQ and should be validated through appropriate experimental studies.

### 2.4. LogP and pK_a_ Value Determination

UV–vis titrations were performed using a V‐670 JASCO spectrophotometer and a Mettler Toledo pH meter. Measurements were carried out in a quartz cuvette (1‐cm path length) maintained at 25.0 ± 0.2 °C. For the determination of the protonation constants, aqueous solutions of LAPQ (4.8–5.0 × 10^−5^ M) were titrated with standardized 0.1 M NaOH. The ionic strength was fixed at 0.1 M with NaCl, and the initial pH was adjusted to 2.6. Each system was investigated through five independent titrations comprising 45–55 data points.

The pKa values of the investigated compounds were initially estimated using three independent spectrophotometric methods, as previously described in the literature [30, 31]. The application of different approaches provided a preliminary evaluation of the acid–base equilibria and allowed cross‐validation of the obtained results. Subsequently, the analysis was performed using the UV–vis spectrophotometric titration datasets processed with the online software KEV: Constant Evaluator [32, 33]. Independently of the initial estimates obtained from the different methods, the fitting procedure consistently converged to the same pKa value, thus confirming the robustness of the adopted equilibrium model. The global analysis allowed refinement of the preliminary estimates through simultaneous fitting of the spectroscopic dataset, reducing the associated uncertainty and improving the precision of the calculated pKa values. The pKa values reported in the manuscript correspond to the mean values obtained from several independent titrations, each analyzed using KEV: Constant Evaluator.

The *n*‐octanol/water partition coefficient (LogP), a useful parameter to evaluate the hydrophilic nature/capability to pass across membranes, was experimentally determined by the shaking‐flask method [34–37]. LAPQ was dissolved in Milli‐Q water at 20 μM and let mix with a defined volume of n‐octanol, the resulting mixture being stirred at 25°C over 3 h. After partitioning, all mixtures were left to equilibrate for at least 60 min. The concentration in the two phases after equilibration was identified using a UV–Vis Cary60 (Agilent Technologies) spectrophotometer (double beam) in the wavelength range of 250–500 nm. The final LogP value, reported as mean ± standard deviation (SD) of three independent measurements, was calculated according to the equation LogP = Log[(A_a_‐A_b_)/A_b_], where A_a_ is the absorbance (proportional to the initial concentration) of the compound in water before partitioning into n‐octanol and A_b_ is the absorbance (proportional to the final concentration) of the compound under examination in water after partitioning at the considered wavelength.

### 2.5. Mass Spectrometry and Study of the Complex

ESI–MS experiments were carried out using a Finnigan LCQ DECA XP PLUS ion trap mass spectrometer (Thermo Electron Corporation, USA), operating in positive ion mode and equipped with an orthogonal ESI source.

Solutions of the metal complexes, diluted with ultrapure water and/or methanol, were introduced into the ion source using nitrogen as the drying gas. The pH of the samples was adjusted to 7 by the addition of NaOH. Copper(II) complexes for ESI–MS analysis were prepared by adding an aqueous solution of the copper ion to an aqueous solution of LAPQ or to a water/methanol 50:50 solution of PQ. Different copper‐to‐ligand molar ratios were examined. The samples were infused into the ion source at a flow rate of 5 μL min^−1^. Instrumental parameters were set as follows: capillary voltage 46 V, capillary temperature 275°C, and spray voltage 4.3 kV. Peak assignments in the ESI–MS spectra were performed using Xcalibur software by comparing the experimental isotopic distributions with the corresponding simulated patterns. Each detected species is reported by the m/z value of the first peak of its isotopic cluster.

The complexation behavior of the newly synthesized ligand LAPQ toward Cu(II) ions was also investigated by UV–vis spectrophotometric titrations. Experiments were carried out at 25°C in a water solution buffered at pH 7.4 with 0.01 M MOPS. UV–vis spectra were recorded during the stepwise addition of Cu(II) solution to a LAPQ solution using a V‐670 JASCO spectrophotometer. The spectral changes observed during the titration were used to evaluate the formation of metal–ligand complexes. The resulting spectroscopic datasets were processed with KEV Evaluator software [32, 33], which allows the simultaneous analysis of the complete set of spectra collected during the titration and the determination of the corresponding conditional stability constants.

### 2.6. CD Spectroscopy

CD measurements were performed using a JASCO J‐1500 spectropolarimeter equipped with a Peltier temperature controller. UV–vis and CD spectra were recorded using cuvettes with a 1 cm optical path length. CD titrations were carried out under the same experimental conditions used for the UV–vis measurements, using LAPQ in 0.01 M MOPS buffer at pH 7.4 upon addition of Cu^2+^ in the range of 0.15–2.25 molar equivalents. Each CD spectrum was obtained by averaging at least five consecutive scans.

### 2.7. SPR Measurements

SPR experiments were carried out on an Octet SF3 system (Sartorius). Aβ_1–40_ peptide dissolved in acetate buffer was immobilized onto a CDL sensor chip (Octet SF3 system, Sartorius) via standard amine coupling chemistry using EDC/NHS as activating reagents [38–40]. The immobilization procedure was performed in three sequential steps. First, the carboxyl groups on active channels and reference channels were activated by injecting a freshly prepared 1:1 mixture of 0.4 M 1‐ethyl‐3‐(3‐dimethylaminopropyl) carbodiimide (EDC) and 0.1 M NHS at a constant flow rate of 10 μL/min for 7 min. Subsequently, 135 μL of Aβ_1–40_ was immobilized at 30 μL/min in acetate buffer (pH 4.2). This pH value, below the isoelectric point of the Aβ peptide (pI = 5.3 [41]), ensures protonation of the peptide and promotes electrostatic attraction to the negatively charged carboxymethyl dextran matrix of the CDL sensor surface, thereby enhancing immobilization efficiency. Finally, residual active esters were quenched and nonspecific adsorption prevented by injecting 1 M ethanolamine (pH 8.5) at 10 μL/min for 420 s, followed by a 60 s dissociation phase. Unbound material was removed by injecting a regeneration solution containing 10 mM NaOH and 1 M NaCl.

The immobilization efficiency of Aβ_1–40_ on the CDL sensor chip was assessed in terms of surface density, yielding an average response of 450 response units (RUs). According to the Sartorius technical documentation, the SF3 instrument reports a response in which 1 RU corresponds approximately to 1 ng/mm^2^ of immobilized protein. This relationship is consistent with general literature values, including those reported by Schasfoort [42], which associate 1 ng/mm^2^ of protein with ∼1000 RU at 670 nm. Considering the molecular weight of 4329.82 Da for full‐length Aβ_1–40_, this corresponds to an estimated surface density of ∼6.26 × 10^10^ molecules per mm^2^.

Both PQ (500, 250, 125, 62.5, 31.3, and 15.6 μM) and LAPQ (750, 375, 187.5, 46.9, and 23.4 μM) samples, prepared by serial dilution in running buffer (10 mM HEPES, 150 mM NaCl, 3 mM EDTA, 0.05% v/v Tween‐20, pH 7.4), were injected at 30 μL/min for 2 min over the immobilized Aβ_1–40_. Evaluation of sensorgrams was performed using Octet SPR Analysis Version 5.0.1 Build 5^©^ 2021 Sartorius BioAnalytical Instruments, Inc.

### 2.8. TEAC Assay

This assay quantifies the antioxidant capacity of test compounds by measuring their ability to scavenge the ABTS^•+^ radical cation (2,2′‐azinobis‐(3‐ethylbenzothiazoline‐6‐sulfonic acid)) relative to Trolox (6‐hydroxy‐2,5,7,8‐tetramethylchroman‐2‐carboxylic acid), a standard antioxidant reference. ABTS^•+^ was produced by mixing ABTS with potassium persulfate in distilled water following the procedure reported elsewhere [43]. The resulting ABTS^•+^ solution was diluted with 5 mM phosphate buffer (pH 7.4) to reach an initial absorbance of 0.70 ± 0.05 at 734 nm. Absorbance at 734 nm was monitored over 6 min using UV–vis spectrophotometry after adding test samples at concentrations from 3 × 10^−6^ M to 3 × 10^−5^ M. A decrease in absorbance indicated scavenging activity of the test compounds on ABTS^•+^, with minimal interference from side reactions. TEAC values were calculated at multiple time points, normalized against Trolox equivalents, and expressed as means ± SD based on triplicate measurements.

### 2.9. AA Assay

The oxidation of AA was monitored by measuring the absorbance at *λ* = 265 nm, which corresponds to the maximum absorption wavelength of reduced AA, in HEPES buffer (50 mM, pH 7.4). HEPES was selected as a buffer system since it does not bind copper ions with significant affinity, thus ruling out its competition as a ligand.

Cu^2+^, the ligand, and AA were added to a cuvette (1 cm path length) to achieve the following final concentrations: [Cu^2+^] = 2.0 μM, [ligand] = 4.0 or 8.0 μM, and [AA] = 120 μM. The kinetics of AA oxidation were monitored, and kinetic parameters were determined from three independent measurements, with freshly prepared solutions used in all instances. The resulting absorbance profiles were used for a qualitative comparison of AA consumption in the presence of AA alone, Cu^2+^, and the Cu(II) complexes of PQ and LAPQ. No absolute oxidation rates or kinetic parameters were calculated. The absorbance contributions of the corresponding copper complexes, measured under identical conditions at 265 nm, were subtracted. Following reduction of the Cu(II) complexes, the released free PQ or LAPQ may also contribute to the absorbance at 265 nm. This contribution would partially compensate for the decrease in absorbance associated with AA consumption and could therefore lead to a slight underestimation of the apparent activity of the corresponding systems. However, this contribution is expected to be limited, particularly during the initial 30 s interval highlighted in the inset, and is not expected to substantially affect the qualitative comparison among the different systems.

### 2.10. Cell Culture

A549 (lung adenocarcinoma, A549/TS2, RRID:CVCL_B7N6) and PC3 (prostate carcinoma cell line, PC3‐MM2, RRID:CVCL_4885) human cancer cell lines were obtained from American Type Culture Collection (Manassas, VA), while HCT116 (colon carcinoma, HCT 116, RRID:CVCL_0291) and HepG2 (hepatoblastoma, A549/TS2, RRID:CVCL_0027) cells were obtained from DIMED, University of Padova (Padova, Italy). A549 and HCT116 were grown in DMEM supplemented with 10% FBS, and PC3 in F‐12K supplemented with 10% FBS, while HepG2 cells were grown in MEM supplemented with 15% FBS and 1% MEM nonessential amino acids. Cells were grown in a humidified incubator at 37°C with 5% CO_2_.

### 2.11. Cell Viability Assays

The compounds under investigation (PQ and LAPQ), along with their combination with Cu^2+^ were evaluated in terms of cytotoxic activity in vitro using the MTT assay. Cells were seeded in 96‐well plates at a density of 1 × 10^4^ cells per well for HCT116 and HepG2 cells and 7 × 10^3^ cells per well for A549 and PC3 cells. Stock solutions of PQ and LAPQ were prepared at a concentration of 10 mM in DMSO and distilled water, respectively. Working solutions at 1 mM were subsequently prepared in water and used for cell treatments.

Cells were treated with PQ or LAPQ, either alone or in combination with 20 μM CuCl_2_, as previously reported [23]. Cells treated with the same volume of the corresponding vehicle were included as negative controls to account for possible solvent‐related effects on cell viability. Cells treated with 20 μM CuCl_2_ alone were also included to distinguish the effect of the copper salt from that of its combination with PQ or LAPQ.

After 72 h of incubation at 37°C, the cells were washed with PBS and incubated with MTT at a final concentration of 0.5 mg mL^−1^ for 3 h at 37°C. Subsequently, 100 μL of stop solution consisting of 90% isopropanol and 10% DMSO was added to each well. After 15 min, absorbance was measured at 595 and 690 nm using an Infinite M Plex microplate reader (Tecan).

### 2.12. Cytoprotective Effect Against an Oxidative Stimulus

HepG2 cells were seeded in 96‐well plates (1 × 10^4^ cells per well) and allowed to adhere for 24 h. Then, cells were treated with increasing concentrations of PQ and LAPQ with or without the addition of 20 μM Cu^2+^. After 6 h, 200 μM TbOOH was added to induce oxidative stress for 18 h. Cell viability was assessed by MTT assay, as reported above. Briefly, cells were incubated with 0.5 mg/mL MTT solution in PBS for 3 h at 37°C. Afterwards, formazan crystals were dissolved in 100 μL of stop solution (90% isopropanol/10% DMSO), and absorbance was read at 595 nm (reference 690 nm). Data were expressed as % viability versus untreated controls.

## 3. Results and Discussion

### 3.1. Synthesis and Characterization

LAPQ was synthesized through an amide coupling reaction between PQ and LA using DCC as the activating agent in the presence of NHS. The reaction led to the formation of the corresponding NHS ester, which subsequently reacted with the amine group of PQ to afford the desired conjugate. Upon completion of the reaction, the precipitated dicyclohexylurea (DCU) by‐product was removed by filtration, providing a first crude purification step. The filtrate was then concentrated and subjected to purification. The purified derivative was characterized by mass spectrometry and NMR spectroscopy. MS analysis confirmed the expected product, with a molecular ion peak adduct [M+H]^+^ at m/z 600.22 consistent with the proposed structure.

The ^1^H NMR and ^13^C NMR spectra clearly demonstrate the formation of the conjugate (Figures S1–S4, SI). Complete signal assignment was achieved through a series of 2D NMR experiments, including HSQC, TOCSY, and HMBC, allowing the identification of proton and carbon atoms within both the PQ and carbohydrate units. The aromatic region between 8.5 and 6.4 ppm displays the characteristic signals attributable to the PQ scaffold, confirming preservation of the heteroaromatic core. Upon amide bond formation, the methylene group α to the nitrogen displays two distinct resonances (*δ* 3.21 and 3.11 ppm), consistent with diastereotopic protons. The partial double‐bond character and resulting conformational restriction of the amide moiety render the two methylene protons magnetically nonequivalent. The other PQ signals remained essentially unchanged when compared to the nonfunctionalized compound. The resonances corresponding to the sugar moiety are observed in the 3.2–4.3 ppm range, as expected for carbohydrate protons. In the ^1^H NMR spectrum (D_2_O), LA moiety shows the characteristic anomeric proton of the galactose unit at 4.30 ppm and is correlated in HSQC with C‐1 at 105 ppm, confirming the β‐glycosidic anomeric center. Other proton signals appear as multiplets in the region between 3.30 and 4.25 ppm attributable to the protons of the gluconic acid and galactose moieties.

We also determined the pK_a_ values of LAPQ, as pKa governs the protonation state of the molecule at different pH values. At physiological pH, the protonation equilibrium of ionizable groups in PQ directly determines which species predominates, and the specific ionization state critically influences the ability of each compound to interact with biological targets, cross biological barriers, and undergo metabolic transformations. The following pK_a_ values for PQ had been experimentally established: pKa_1_ = 3.20 and pKa_2_ = 10.39 [44]. These two distinct ionization constants have been attributed to the stepwise protonation of the heterocyclic quinolinic nitrogen atom and to the primary amine nitrogen located along the aliphatic side chain (corresponding to pKa_1_ and pKa_2_). As for LAPQ, a single acid ionization constant (pK_a_ = 3.92 ± 0.06) was determined spectroscopically and assigned to the protonation of the heterocyclic quinoline nitrogen (Figure S5–S8, SI). This finding is consistent with the structural modification of LAPQ, in which the primary amine was converted into an amide. As a result, its pK_a_ falls outside the range accessible by the employed method. The species distribution diagram indicates that, at physiological pH, LAPQ is predominantly present in its neutral form, whereas PQ, which exhibits a pK_a_ of approximately 10.39 for its aliphatic amine, is almost entirely protonated under the same conditions and therefore exists mainly as a monocationic species.

The experimental LogP values for PQ have been consistently documented across multiple studies and computational platforms. Experimental determinations have yielded a LogP value of approximately 2.1 for PQ, whereas an experimental LogD_7_._4_ value of 0.54 has been reported under physiological conditions [45, 46]. In this work, we experimentally determined the LogP value of LAPQ to be 0.09 ± 0.04. Since LAPQ is almost entirely present in its neutral form at physiological pH (Figure S8), its LogD_7_._4_ is expected to be very close to its experimentally determined LogP (LogD ≈ LogP). Therefore, when compared to the higher LogP value of 2.1 and, more appropriately, under physiological conditions, to the LogD_7_._4_ value of 0.54 reported for PQ, this result clearly highlights the increased hydrophilic nature of LAPQ. The value, close to zero, indicates an almost equal partition between the aqueous and organic phases. The lower lipophilicity of LAPQ compared to PQ accounts for the pronounced aqueous solubility of the compound, thus enabling all subsequent experiments to be performed exclusively in water or buffered media, eliminating the need for organic co‐solvents.

The in silico ADME evaluation of LAPQ highlighted a pharmacokinetic profile strongly influenced by the presence of the LA moiety (Table S1, SI). The compound exhibited low lipophilicity (predicted LogP = 0.104), in good agreement with the experimentally determined value (experimental LogP = 0.09), and a high topological polar surface area (TPSA = 243.55 Å^2^), consistent with its enhanced aqueous solubility compared to PQ. These features, together with the high number of hydrogen‐bond donors and acceptors, are predicted to limit passive membrane diffusion, as reflected by the low Caco‐2, MDCK, and PAMPA permeability values. Furthermore, LAPQ was predicted to be a P‐glycoprotein substrate, suggesting the possibility of active efflux processes. Despite its limited permeability, LAPQ showed acceptable plasma protein binding (69.9%) and a relatively high unbound fraction in plasma (29.4%). Notably, the compound was predicted not to cross the blood–brain barrier, a result consistent with its high polarity and molecular size. The metabolic profile suggested a low propensity to inhibit most cytochrome P450 isoforms, although interactions with CYP2C8 and metabolism mediated by CYP2C19 and CYP3A4 cannot be excluded. Additionally, the predicted stability in human liver microsomes indicates that the molecule may possess adequate metabolic stability.

Overall, the ADME predictions indicate that conjugation of PQ with LA successfully generated a more hydrophilic and less membrane‐permeable derivative. While these characteristics may limit oral bioavailability and central nervous system exposure, they could be advantageous for reducing nonspecific tissue distribution and toxicity, in agreement with the lower cytotoxicity experimentally observed for LAPQ.

### 3.2. Interaction of the Compounds With Aβ Peptide

It is noteworthy that the repurposing of PQ goes beyond its classical role as an antimalarial drug, suggesting potential relevance in amyloid disorders and other conditions involving protein aggregation. Studies have shown that PQ can reduce protein aggregates [47]. It has also been explored in the design of antiamyloid agents targeting polyanionic glycosaminoglycans [48] and in interaction studies with model proteins such as bovine serum albumin [49].

**FIGURE 2:**
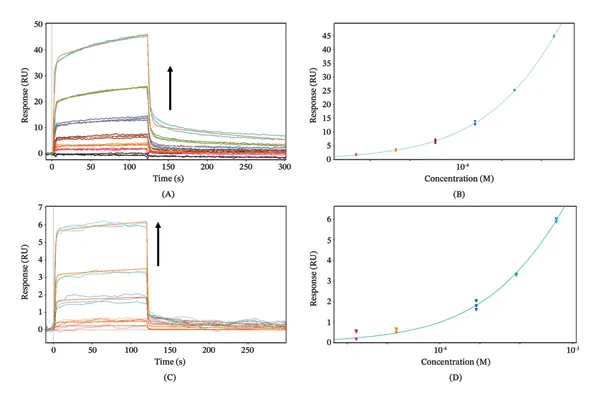
(A) Sensorgram of the interaction between PQ and immobilized Aβ_1–40_ on the CDL sensor chip, showing increasing SPR response (RU) with rising PQ concentrations. Data were fitted to a 1:1 two‐state binding model (red lines). (B) Dose–response plot: Equilibrium SPR responses (RU) at the sensorgram plateau (100–120s) were plotted against PQ concentration. (C) Sensorgram of the interaction between LAPQ and immobilized Aβ, showing increasing SPR response (RU) with rising LAPQ concentrations. Data were fitted to a 1:1 two‐state binding model (red lines). (D) Dose–response plot: Equilibrium SPR responses (RU) at the sensorgram plateau (100–120s) were plotted against LAPQ concentration.

PQ has been reported to inhibit human erythrocyte acetylcholinesterase (AChE) [50]. Although AChE inhibitors are primarily used in the management of AD to counteract cholinergic deficits associated with neuronal loss [49], cholinergic signaling is not restricted to the central nervous system and may also contribute to physiological and pathological processes in peripheral tissues [51]. Therefore, the AChE inhibitory activity of quinoline derivatives may support their potential repositioning not only in AD but also in disorders characterized by oxidative stress and Aβ accumulation. In this context, compounds combining cholinesterase inhibition with antioxidant properties may offer additional therapeutic value. Despite the potential relevance of PQ in Aβ‐related disorders, no information is currently available regarding its direct interaction with the Aβ peptide. To address this gap, we performed an SPR study, enabling real‐time, label‐free, and quantitative characterization of the PQ–Aβ interaction. As shown in Figure 2, the interaction between PQ and Aβ generates positive SPR responses that increase progressively with rising analyte concentration.

The kinetic parameters of the interaction were determined by applying the 1:1 two‐state binding model, which accounts for conformational changes of the ligand upon interaction (Octet SPR analysis, Sartorius), thereby enabling calculation of the corresponding dissociation constant (K_D_) that quantifies the binding affinity, as lower K_D_ values indicate stronger ligand–analyte interactions. In parallel, a dissociation constant was obtained from the dose–response analysis (Figure 2), in which the maximum SPR responses (RU) at steady state were plotted against the corresponding PQ concentrations. Both approaches indicated that the PQ–Aβ interaction yields an average K_D_ value of 1.15 ± 0.41 mM.

The same two‐state binding model was applied to the LAPQ–Aβ interaction, enabling determination of the kinetic parameters and calculation of the corresponding K_D_. This analysis revealed an average K_D_ value of 2.04 ± 0.56 mM. This is a twofold value compared to the PQ–Aβ interaction that corresponds to a relatively small change in stability, typically amounting to only a few kJ·mol^−1^ in free energy.

### 3.3. Copper Complexes

The formation of coordination compounds between copper and the two organic ligands, PQ and LAPQ, was investigated in solution under conditions closely mimicking the physiological environment. A percentage of organic solvent was required to avoid precipitation in the case of PQ. Positive‐ion mode ESI–MS spectra of solutions containing PQ and Cu^2+^ (see Figures S9–S11, SI) revealed the formation of copper–ligand species. When Cu^2+^ was reacted with PQ at a 1:1 metal‐to‐ligand molar ratio in a 50:50 water/methanol mixture (pH ≈ 7), the spectra indicated the formation of 1:1 metal–ligand complexes. In addition to the pseudo‐molecular ion [M + H]^+^ of PQ at 260.2 m/z, characteristic copper‐containing cluster peaks were observed at 321.1 and 357.1 m/z. The signal at m/z 321.1 was assigned to a CuL species (Table 1), consisting of one PQ ligand coordinated to a copper ion. This attribution is strongly supported by the characteristic copper isotopic envelope. Specifically, the experimental isotopic pattern and the relative peak intensities are not consistent with a single ionic species but rather indicate the partial overlap of two closely related ions, namely [Cu^I^L]^+^ and [Cu^II^L–H]^+^. The superposition of these two species, which have m/z values that differ by one, accounts for the observed peak ratios within the isotopic cluster. Although Cu^2+^ was initially present in solution, partial reduction to Cu^+^ is a well‐documented phenomenon under ESI–MS conditions. Such gas‐phase redox processes frequently occur during the ionization and desolvation steps and have been widely reported in the case of copper complexes [52]. The signal at m/z 351.1 can also be attributed to a CuL species where the metal coordination sphere is completed by the binding of two water molecules (see Table 1). As for LAPQ, the ESI spectrum in water solution (pH ≈ 7) highlights the presence of a peak at 661.4 m/z corresponding, also in this case, to a complex species with a 1:1 metal‐to‐ligand stoichiometry. In addition, a species with a Cu_2_L stoichiometric ratio is observed at very low relative intensity. This signal may arise from the association of a second Cu(II) ion with additional donor sites of LAPQ, possibly involving the deprotonated amide nitrogen and/or oxygen donors of the lactobionic moiety. However, since this species was detected by ESI–MS, it may also represent a low‐abundance metal adduct formed during the electrospray ionization process rather than a significantly populated species in solution.

**TABLE 1:** ESI–MS characterization of the Cu^2+^ complexes of PQ and LAPQ.

Sample	Species	Assignment	Calcd. (m/z)	Exp. (m/z)	RI (%)^a^
PQ/Cu 1:1	[PQ + H]^+^	C_15_H_22_N_3_O	260.3	260.2	100%
	[PQ + Cu^2+^−H]^+^	C_15_H_20_N_3_OCu^II^	321.1	321.1	35%
	[PQ + Cu^+^]^+^	C_15_H_21_N_3_OCu^I^	322.1	322.1	
	[PQ + Cu^2+^ + 2H_2_O −H]^+^	C_15_H_24_N_3_O_3_Cu^II^	357.1	357.1	15%

LAPQ/Cu 1:1	[LAPQ + H]^+^	C_27_H_42_N_3_O_12_	600.3	600.4	80%
	[LAPQ + Na]^+^	C_27_H_41_N_3_O_12_Na	662.3	622.6	100%
	[LAPQ + Cu^2+^ − H]^+^	C_27_H_40_N_3_O_12_Cu	661.2	661.4	25%
	[LAPQ + 2Cu^2+^ − 3H]^+^	C_27_H_38_N_3_O_12_Cu_2_	722.1	722.3	5%

^a^Relative intensity.

**FIGURE 3:**
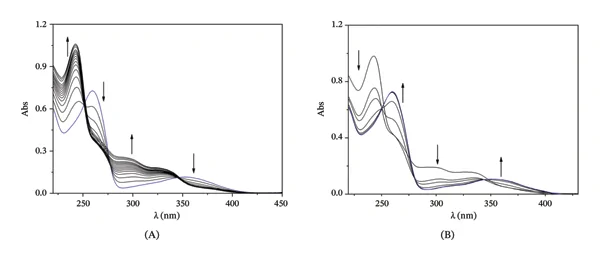
(A) UV–vis titration of LAPQ (4.9 × 10^−5^ M) upon addition of Cu^2+^ (0–2.2 mol equiv, 0.2 mol equiv increments) in 0.01 M MOPS at pH 7.4. (B) UV–vis titration of LAPQ–Cu^2+^ complexes with increasing concentrations of GSH (0–2 mol equiv, 0.5 mol equiv increments) at pH 7.4 in 0.01 M MOPS buffer; the spectrum of LAPQ alone is reported in blue.

The formation of copper complex species of PQ and LAPQ was also monitored by UV–vis and CD spectroscopy. The spectrum of PQ in MOPS/dioxane 70:30 shows two bands at 264 and 358 nm (see Figure S12). The intensity of these two bands decreased with increasing copper concentration, suggesting the binding. The spectrum of LAPQ recorded in MOPS shows two intense bands at 260 nm and 353 nm, corresponding to π–π∗ and n–π∗ transitions (Figure 3A). Following the titration of LAPQ with a Cu^2+^ solution, the peaks at 260 and 353 nm significantly diminished, and new bands emerged at 242, 296, and 332 nm. CD spectroscopy was also used to investigate the conformational and electronic changes associated with Cu^2+^ coordination. Although LAPQ contains a chiral LA moiety, the free ligand shows no appreciable CD signal in the investigated wavelength range, suggesting that the electronic transitions of the quinoline chromophore are not significantly influenced by the chiral portion in the uncomplexed, conformationally flexible molecule. The CD spectrum of the Cu^2+^–LAPQ system shows the appearance of two negative bands at 246 nm (with a shoulder at 269 nm) and 328 nm (see Figure S13, SI). The emergence of these CD bands provides additional evidence of complex formation and of the conformational reorganization induced by Cu^2+^ coordination. The mole ratio plot of UV data shows a change in slope at approximately M/L ≈ 0.5, consistent with the formation of a 1:2 (ML_2_) complex. However, the lack of a clear, sharp inflection point suggests the system does not undergo a complete, abrupt conversion to a single dominant species (see Figure S14, SI). Rather, the data imply that multiple species, most likely ML and ML_2_, coexist in solution over the tested concentration range. This gradual shift aligns with stepwise formation constants of comparable magnitude and/or moderate overall complex stability, leading to a slow redistribution of species rather than a sharp stoichiometric break. The analysis of the spectrophotometric titration data collected in aqueous solution using the online KEV Evaluator software allowed the determination of the cumulative conditional stability constants of the Cu(II)–LAPQ complexes. The fitting of the UV–vis data according to an appropriate equilibrium model revealed the formation of both ML and ML_2_ species, in agreement with the stoichiometries detected by the molar ratio method and ESI–MS, with cumulative conditional stability constants of log β_1_ = 4.4 ± 0.3 and log β_2_ = 8.7 ± 0.1. On the basis of the speciation and spectroscopic data, together with literature reports on PQ metal complexes [26, 27], a coordination model can be proposed for LAPQ. In the mononuclear CuL species, Cu(II) is coordinated through the N,N donor set of the 8‐AQ moiety of LAPQ, while the remaining coordination sites are occupied by water or may also involve deprotonated amide nitrogen or additional oxygen donors from the conjugate. For the CuL_2_ species, coordination can occur through the N,N donor sets of two 8‐AQ moieties, resulting in a bis‐chelated coordination environment around the Cu(II) ion. This coordination mode is consistent with models previously reported for Cu(II) complexes of PQ [26].

### 3.4. Interaction With GSH

Glutathione (γ‐glutamyl–cysteinyl–glycine) represents the predominant low‐molecular‐weight thiol in mammalian cells, where it is typically present at millimolar concentrations. The reduced glutathione/oxidized glutathione (GSH/GSSG) redox couple plays a pivotal role in maintaining cellular redox balance. Under physiological conditions, glutathione exists mainly in its reduced form and participates in multiple biological processes, including regulation of protein thiol oxidation, protection against oxidative stress, and detoxification of metal ions [53–55].

Glutathione can act as an intracellular reducing agent toward Cu^2+^ ions, and even trace amounts of copper are sufficient to promote GSH oxidation to GSSG. In this context, an interaction between the Cu^2+^–PQ/LAPQ complex and GSH is highly plausible. Therefore, we investigated the nature of this interaction.

The reaction between the Cu^2+^–LAPQ complex and GSH was spectroscopically monitored under aerobic conditions at pH 7.4 (Figure 3B). Upon addition of one equivalent of GSH, a rapid decrease in the absorption bands at 243, 296, and 332 nm was observed, together with a concomitant increase in absorbance at 260 nm. These spectral changes indicate a reduction in the concentration of the Cu^2+^–LAPQ complex accompanied by the release of the free ligand. More specifically, GSH reduces Cu(II) and is concomitantly oxidized to GSSG; the resulting GSSG then coordinates the copper center, replacing the original ligand in its coordination sphere.

The addition of two equivalents of GSH resulted in complete copper dissociation from the original Cu^2+^–LAPQ complex. Notably, in the wavelength region where GSH and GSSG and their Cu complexes do not significantly absorb, the UV–vis spectrum of the LAPQ–Cu^2+^–GSH mixture (1:1:2) closely overlapped with that of free LAPQ at the same concentration, confirming that LAPQ had released the metal ion. CD measurements confirmed this behavior (see Figure S15, SI), which was likewise observed for PQ. In a previous study, we demonstrated that the 8‐AQ ligand releases Cu(I), which is subsequently sequestered by GSSG, supporting a mechanistic similarity between the two systems [23].

Taken together, these results indicate that, in the intracellular environment, copper release from the complexes is likely to occur under the reducing conditions characteristic of the cell, probably via GSH interaction.

### 3.5. Free Radical Scavenging Activity

**FIGURE 4:**
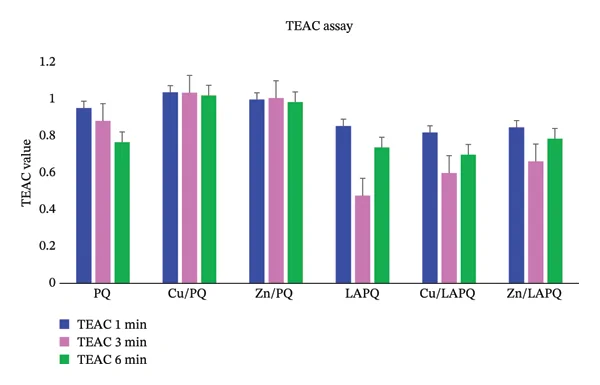
TEAC values at 1, 3, and 6 min for PQ and LAPQ with and without the addition of metal ions (Cu^2+^ and Zn^2+^) in MOPS (10 mM, pH 7.4). TEAC values are the mean ± SD of 3 experiments.

The ABTS^•+^ free radical scavenging assay is one of the most used methods for measuring antioxidant activity in the lab. It was chosen because it is quick, sensitive, and versatile, working well in both water‐based and mixed solvents to evaluate compounds with different polarities like PQ and LAPQ. This assay detects how well compounds can donate hydrogen atoms or electrons, with antioxidant strength expressed as TEAC values at specific time points [56]. TEAC values are calculated by comparing the radical scavenging activity of the tested compound to that of Trolox (a water‐soluble vitamin E analog) under identical experimental conditions, expressed as a ratio relative to Trolox activity, where a value of 1 indicates antioxidant activity equivalent to Trolox at the same concentration. The reduction kinetics of ABTS^•+^ reduction have been widely characterized, and antioxidant behavior is known to depend on several experimental variables, particularly pH, reaction time, temperature, and the molar ratio of ABTS radical cation to the test compound, all influencing the TEAC calculations. For this reason, experiments were carried out under constant temperature and at a fixed pH of 7.4 in order to minimize external variables. Additionally, we evaluated the radical scavenging activity of PQ and LAPQ in the presence of metal ions. Our studies, shown in Figure 4, indicate that both PQ and LAPQ chelate copper with moderate affinity, whereas they do not appear to bind zinc to a significant extent (see Figure S16, SI). Both PQ and LAPQ exhibited TEAC values of approximately 1, indicating a radical scavenging activity comparable to, or slightly lower than, that of Trolox. The presence of either copper or zinc did not negatively affect this antiradical activity; in some cases, a slight enhancement was observed. These findings suggest that the PQ moiety itself is primarily responsible for the observed antiradical effect. The radical‐scavenging activity of PQ is likely mediated predominantly by an electron‐transfer mechanism rather than hydrogen‐atom donation, consistent with its 8‐AQ structure.

PQ is not a classical phenolic antioxidant, but it is a redox‐active compound whose biological activity is often associated with the generation of ROS and oxidative stress. Consequently, while PQ may exhibit limited or context‐dependent direct radical‐scavenging activity in chemical assays such as the TEAC assay, this does not reflect its predominant pro‐oxidant effects in cellular systems. Therefore, TEAC results obtained with PQ should be interpreted cautiously, as chemical antioxidant capacity does not necessarily correlate with its biological mechanism of action, which largely involves oxidative stress–mediated cytotoxicity and apoptosis.

### 3.6. AA Assay of PQ and LAPQ

**FIGURE 5:**
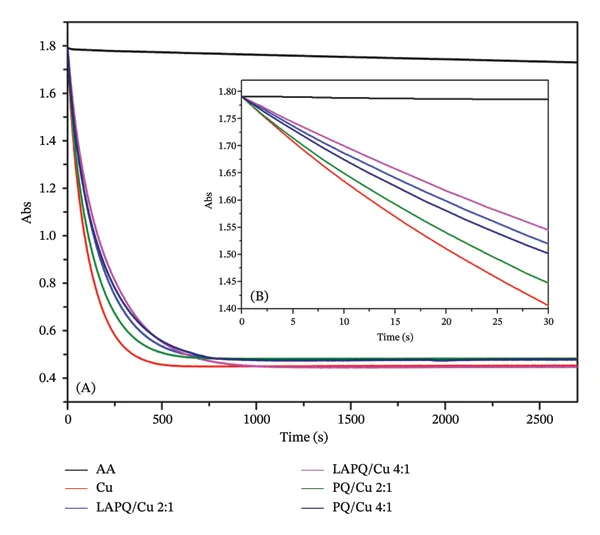
(A) Representative kinetics of ascorbate (AA) consumption, measured by UV–vis at 265 nm, in the presence of AA alone, Cu^2+^−AA (Cu), and Cu complexes of PQ and LAPQ. (B) Magnification of the first 30 s. Conditions: AA 100 μM, Cu^2+^ 2 μM, ligand 4 or 8 μM, MOPS 20 mM pH 7.4, cuvette path length of 1 cm.

The redox activity of copper complexes formed with PQ and LAPQ was evaluated using the AA oxidation assay, a well‐established method to assess copper‐mediated ROS generation (Figure 5). AA was quite stable in the absence of Cu ions in HEPES at pH 7.4, as suggested by the trend of absorbance at 265 nm. Free Cu^2+^ rapidly oxidized AA, as suggested by the rapid decrease of the absorbance at 265 nm, consistent with its efficient redox cycling between Cu^2+^ and Cu^+^ and its ability to promote Fenton‐like reactions leading to the formation of highly reactive hydroxyl radicals (•OH).

Both Cu–PQ and Cu–LAPQ complexes showed a notably lower rate of AA oxidation compared to free Cu^2+^, with LAPQ being particularly more effective in inhibiting oxidation. Reduced AA consumption indicates a significant decrease in copper redox cycling when bound to either ligand, leading to an expected reduction in hydroxyl radicals that is expected to be substantially decreased in the presence of the complexes.

These findings suggest that coordination of copper by LAPQ effectively modulates its redox activity, likely through stabilization of the metal center and/or restriction of its access to reducing agents like AA. The diminished pro‐oxidant activity of these complexes compared to free Cu^2+^ highlights their potential to mitigate copper‐induced oxidative stress. Overall, the AA assay confirms that LAPQ significantly reduces the redox‐driven generation of ROS by copper, supporting their potential as redox‐modulating chelators for biological applications.

### 3.7. Evaluation of Cellular Protection Against Oxidative Stress

**FIGURE 6:**
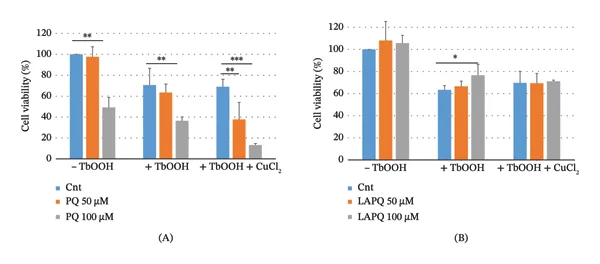
Effect of PQ (A) and LAPQ (B) as cytoprotective agents. HepG2 cells were treated with the quinolines at 50 or 100 μM final concentration and, after 6 h, exposed to 200 μM TbOOH. After 24 h from the quinoline addition, cell viability was assessed by the MTT assay as reported in the Materials and Methods section. The graphs show the mean ± SD of 4 biological replicates, ^∗^ *p* < 0.05, ^∗∗^ *p* < 0.01, ^∗∗∗^ *p* < 0.001.

The protective effect of PQ and LAPQ against oxidative stress was evaluated using TbOOH as an exogenous ROS inducer, followed by assessment of cell viability using the MTT assay. As shown in Figure 6, exposure of cells to TbOOH resulted in a marked reduction in cell viability, confirming the induction of oxidative damage.

Pretreatment with the PQ or with its Cu^2+^ complex did not protect cells from TbOOH‐induced cytotoxicity; instead, a synergistic reduction in cell viability was observed. Indeed, at higher concentrations, PQ significantly enhanced the cytotoxic effect of TbOOH, both in the absence and in the presence of copper. This outcome is consistent with the expected pro‐oxidant behavior of PQ. On the contrary, LAPQ alone significantly attenuated TbOOH‐induced cytotoxicity at the highest concentrations. The improvement in MTT reduction suggests that LAPQ preserves metabolic activity under oxidative stress conditions.

Importantly, the protective effect observed is consistent with the antioxidant activity in vitro of LAPQ and the absence of cytotoxicity.

### 3.8. Antiproliferative Activity

The antiproliferative activity of PQ and LAPQ was evaluated in vitro toward four human cancer cell lines, namely A549 (non–small‐cell lung cancer), HCT116 (colorectal carcinoma), HepG2 (hepatocellular carcinoma), and PC3 (prostate cancer). The compounds were tested both alone and in the presence of Cu^2+^ (20 μM) (Table 2). PQ displayed an intrinsic cytotoxic activity across the investigated models, appearing more active toward the A549 cell line, where the EC_50_ value was around 10 μM. In HepG2, PC3, and HCT116 cells, slightly higher EC_50_ values (40–60 μM) were observed. In the latter, the addition of copper slightly increases the activity. Nevertheless, this effect was modest compared to what we previously reported for the parent 8‐AQ compound (Figure 1), for which the interaction with copper doubled the antiproliferative activity [23], as well as for other 8‐HQ–based systems, known to act through a metal‐ionophore mechanism [24, 57]. The bioconjugate LAPQ was substantially less active, as no significant cytotoxicity was observed (EC_50_ > 100 μM), either in the absence or presence of copper.

**TABLE 2:** EC_50_ values (μM ± SD) of PQ and LAPQ in the absence and presence of a constant Cu(II) concentration of 20 μM for each human cancer cell line.

System	Cell line			
	A549	HCT116	HepG2	PC3
PQ	9.3 ± 0.8	58.9 ± 4.1	47.1 ± 3.2	42.5 ± 1.8
Cu^2+^–PQ	13.5 ± 0.6	44.9 ± 3.6	34.7 ± 4.7	27.6 ± 2.3
LAPQ	> 100	> 100	> 100	> 100
Cu^2+^–LAPQ	> 100	> 100	> 100	> 100
Cu^2+^	> 50	> 50	> 50	> 50

*Note:* Values are reported as the mean of at least three independent experiments after 72 h of incubation.

Overall, these results point out that PQ mainly inhibits cell growth through its intrinsic pharmacological activity rather than via a copper‐mediated ionophore mechanism. PQ is known to exert its anticancer activity through a dual mechanism involving redox imbalance and disruption of endosomal trafficking [58]. It induces ROS and cellular stress, ultimately promoting apoptosis in tumor cells [59]. At the same time, it interferes with endosomal dynamics and receptor trafficking, leading to the downregulation of key oncogenic signaling pathways [58, 60]. The slight increase in antitumor effect observed on HCT116, PC3, and HepG2 cells in the presence of copper suggests a much weaker affinity of PQ for copper compared to 8AQ [23] and implies a different mechanism of action where metal binding may serve as a minor modulator rather than the main cause of cell toxicity.

## 4. Conclusion

In this work, we performed a comprehensive evaluation of compounds PQ and LAPQ, considering the potential relevance of the PQ scaffold in both neurodegenerative and cancer‐related contexts. Their copper‐coordination ability, interaction with Aβ, antioxidant properties, and capacity to modulate oxidative stress were investigated alongside their antiproliferative activity. Both PQ and LAPQ demonstrated a moderate copper‐binding ability. Notably, the AA oxidation assay showed that copper coordination by LAPQ significantly attenuated redox cycling compared with free Cu^2+^, resulting in reduced formation of highly ROS. Under oxidative stress conditions induced by TbOOH, LAPQ also exerted a protective effect at higher concentrations, thereby preserving cell viability. These findings underline the importance of controlling copper reactivity and oxidative damage in biological environments. At the same time, the antiproliferative assays revealed distinct profiles for the two compounds. PQ displayed intrinsic cytotoxicity and a more pronounced antiproliferative effect, only slightly influenced by the presence of copper. In contrast, LAPQ showed markedly improved biocompatibility, no detectable cytotoxicity, and greater aqueous solubility compared with PQ, representing a clear structural and pharmacological advantage. These properties may account for its more controlled biological behavior and reduced nonspecific cellular damage. The interaction of PQ and LAPQ with Aβ further strengthens the significance of these findings. Such binding is particularly relevant because Aβ aggregation is a central event in degenerative pathologies, and small molecules capable of directly interacting with the peptide may influence its conformational behavior and aggregation propensity. Therefore, beyond their metal‐coordinating and redox‐modulating properties, PQ and especially LAPQ may exert an additional mechanism of action through direct modulation of Aβ. Moreover, oxidative stress is a central contributor to Aβ–induced toxicity, as its aggregation promotes ROS production, lipid peroxidation, mitochondrial dysfunction, and cellular damage [61]. Therefore, the antioxidant effect shown here for LAPQ might contribute to mitigating Aβ‐mediated injury and slowing disease progression.

Overall, LAPQ emerges as a multifunctional compound, capable of modulating copper homeostasis and oxidative stress with a more favorable safety profile. Overall, the combined anti‐amyloid and antioxidant properties of LAPQ identify this compound as a promising starting point for the development of therapeutic approaches targeting Aβ‐related disorders, particularly in settings where peripheral rather than central activity is desirable.

## Author Contributions

Alessia Distefano performed the UV–vis titration experiments, conducted the SPR studies, and carried out the TEAC assay.

Laura Cifalinò conducted selected titration experiments and CD studies, evaluated the interaction with GSH, performed the ascorbate assay, and carried out mass spectrometry analyses.

Giuseppe Grasso supervised the SPR studies and contributed to the writing of the manuscript.

Alessia Sambugaro and Erik Murador carried out the cytotoxicity assays on A549 and PC3 cells as well as LogP evaluations.

Alessandra Folda carried out the cytotoxicity assays on HepG2 and HCT116 cells.

Maria Pia Rigobello supervised the research activities and acquired the funding.

Chiara Nardon acquired the funding, supervised some research activities, and contributed to the physicochemical characterization and manuscript editing.

Valeria Scalcon carried out the cellular antioxidant assays on HepG2 cells and contributed to manuscript preparation.

Valentina Oliveri conceived and designed the study, supervised the research activities, acquired the funding, and performed the chemical synthesis, purification, and full characterization of the compounds.

## Funding

This research was supported by the Ministry of the University and Research: PRIN2022 project (n. 2022BTMYWZ; CUP code: B53D23015260006) entitled “New mEtal‐baSed agenTs against Orphan tumoRs–NESTOR” [announcement D.D. 104 del 02/02/2022; PNRR per la Missione 4, Componente 2, Investimento 1.1], financed by the “European Union–NextGenerationEU.” Alessia Distefano, Giuseppe Grasso, and Valentina Oliveri thank the ERMES project, funded under the European Union’s Horizon Europe EIC Pathfinder Open programme, grant agreement no. 101185661. Giuseppe Grasso and Valentina Oliveri also thank the Italian Ministry of Health: Piano di Sviluppo e Coesione del Ministero della Salute 2014–2020, Project: Pharma‐HUB—Hub per il riposizionamento di farmaci nelle malattie rare del sistema nervoso in età pediatrica (CUP E63C22001680001; ID T4‐AN‐04). Valentina Oliveri and Laura Cifalinò also thank Piaceri2024‐TRACE. Chiara Nardon thanks Martina Russo, Giorgia Capparelli, and Rachele Bonzanini as well as Dr. Serena Zanzoni (Facility “Centro Piattaforme Tecnologiche” [CPT]) for technical support. The authors gratefully acknowledge Dr. George Gamov (Ivanovo State University of Chemistry and Technology) for providing access to and support in the use of the KEV Evaluator software, which was employed in this study.

## Conflicts of Interest

The authors declare no conflicts of interest.

## Supporting Information

Additional supporting information can be found online in the Supporting Information section.

## Supporting information


**Supporting Information** The Supporting Information is available and includes additional spectroscopic and analytical data supporting the characterization and solution studies of the investigated systems. In particular, it contains the ^1^H NMR, HSQC, TOCSY, and APT spectra of LAPQ (Figures S1–S4), UV–vis and acid‐base titration experiments together with the calculated molar absorptivity spectra of the protonated and neutral forms and species distribution diagrams (Figures S5–S8), ESI–MS spectra and isotopic pattern simulations for the PQ/Cu^2+^ and LAPQ/Cu^2+^ systems (Figures S9–S11), UV–vis titrations of PQ upon addition of Cu^2+^ with the corresponding absorbance plots versus metal/ligand molar ratio (Figures S12), CD titration experiments, molar ratio graphs, and CD spectra of LAPQ and related mixtures with Cu^2+^ and glutathione (Figures S13–S15), as well as UV–vis titration data of LAPQ in the presence of Zn^2+^ (Figure S16). Table S1. In silico ADMET analysis of LAPQ.

## Data Availability

The data that support the findings of this study are available in the supporting information of this article.
