# Supplementary material for: Lactobionic Acid as a Biocompatibility Switch: Physicochemical and Biological Characterization of Copper(II)–Primaquine Complexes
Source: Bioinorg Chem Appl. 2026 Sep 9;2026:9997326. doi: 10.1155/bca/9997326 (PMC13559010; doi:10.1155/bca/9997326)
Supplement: Supplementary file 1 [file BCA-2026-9997326-s001.pdf]

Supporting Information

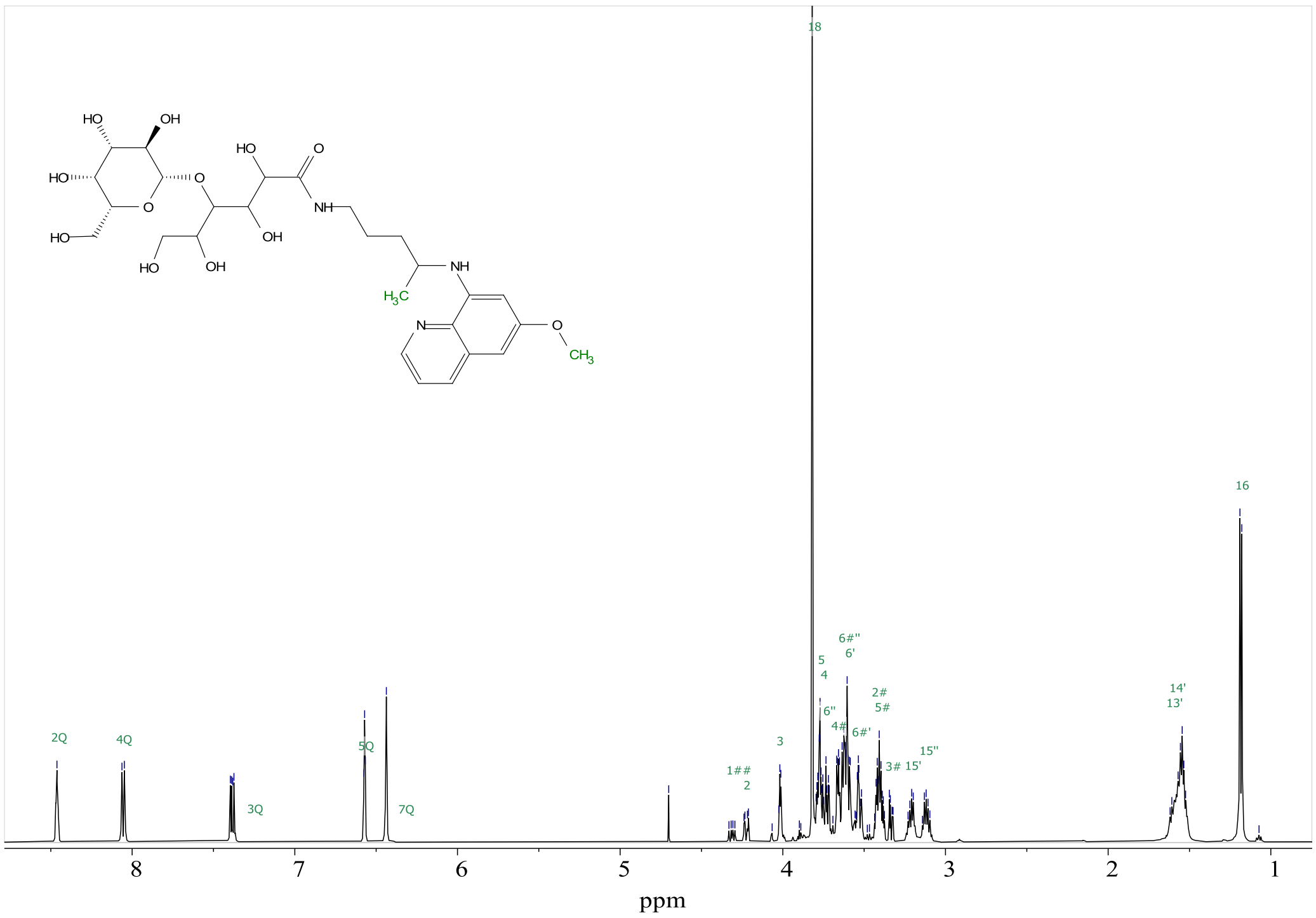

Figure S 1. ***^{1}H NMR spectrum of LAPQ in D_2O at 600 MHz.***

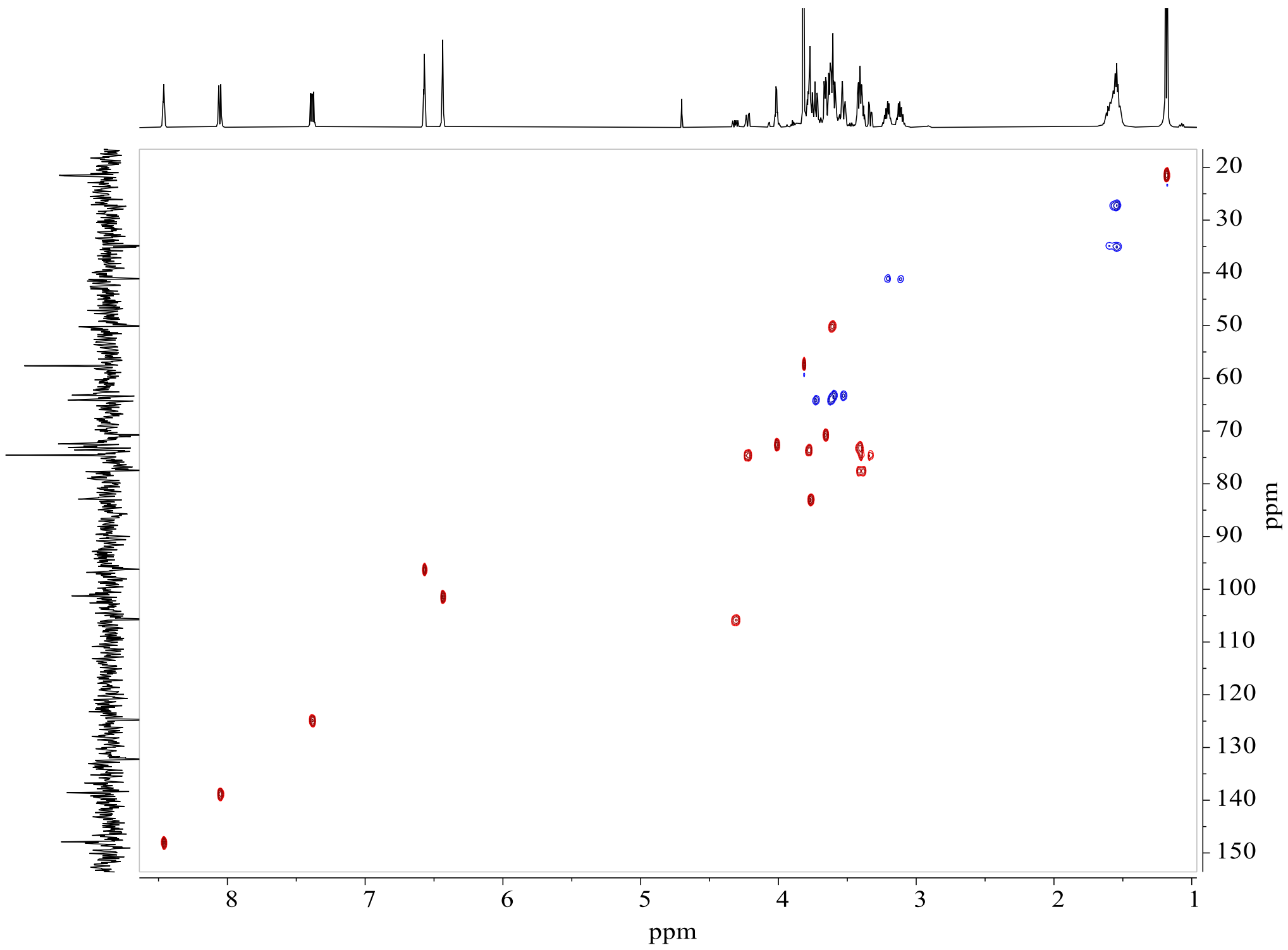

Figure S 2. HQSC spectrum of LAPQ in D_2O at 600 MHz.

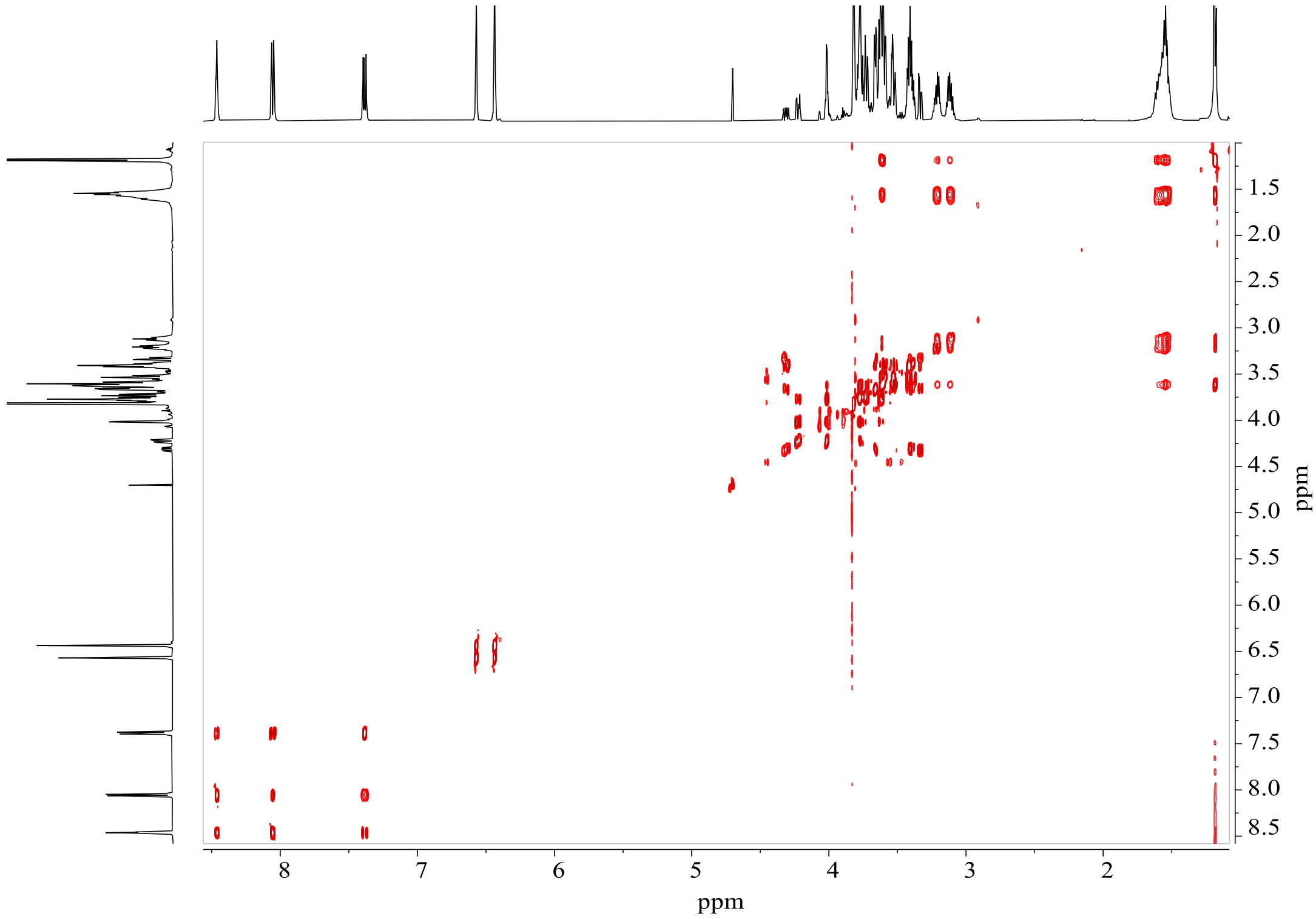

Figure S3.TOCSY spectrum of LAPQ in D_2O at 600 MHz.

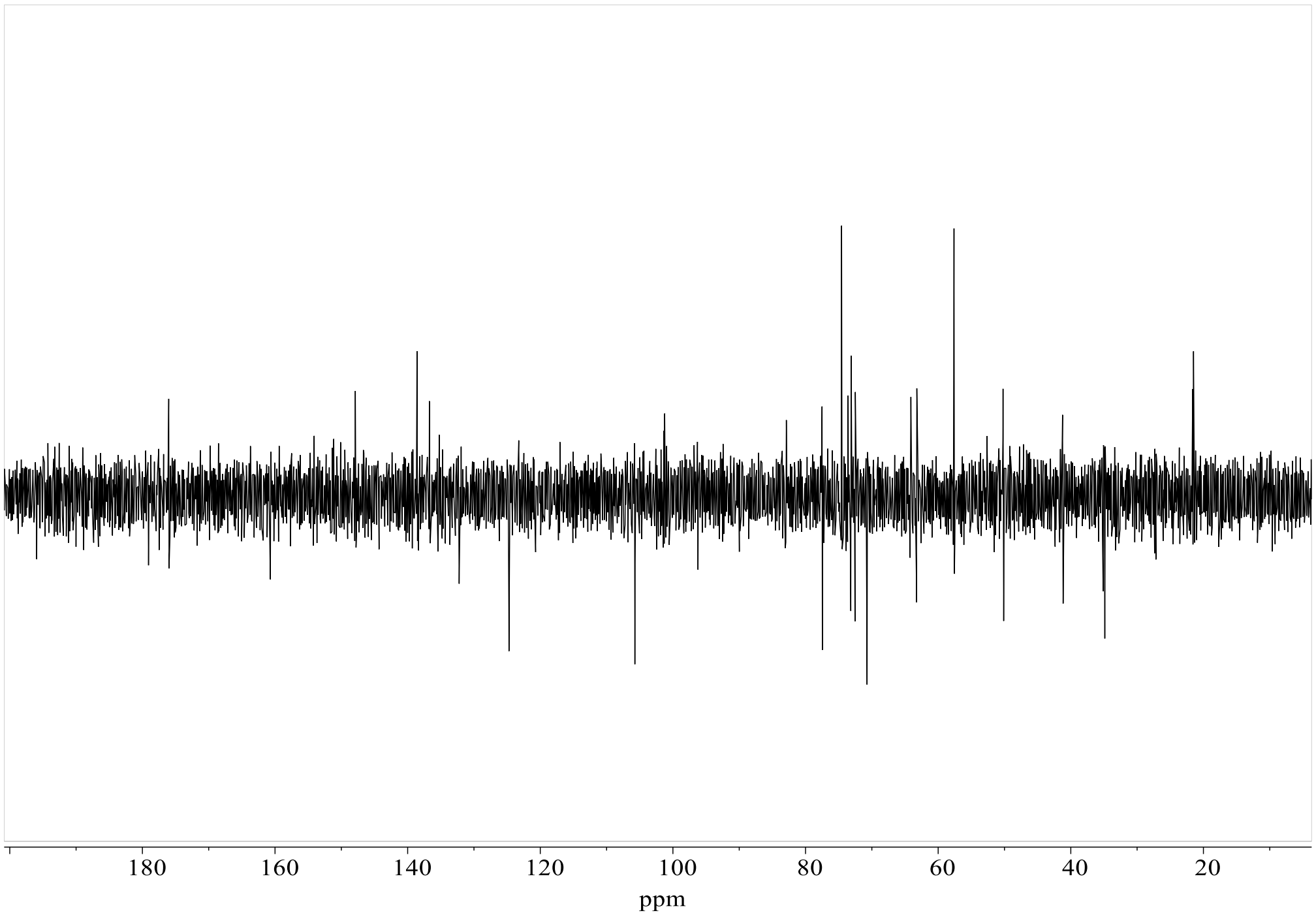

Figure S4. Apt spectrum of LAPQ in D_2O at 150 MHz.

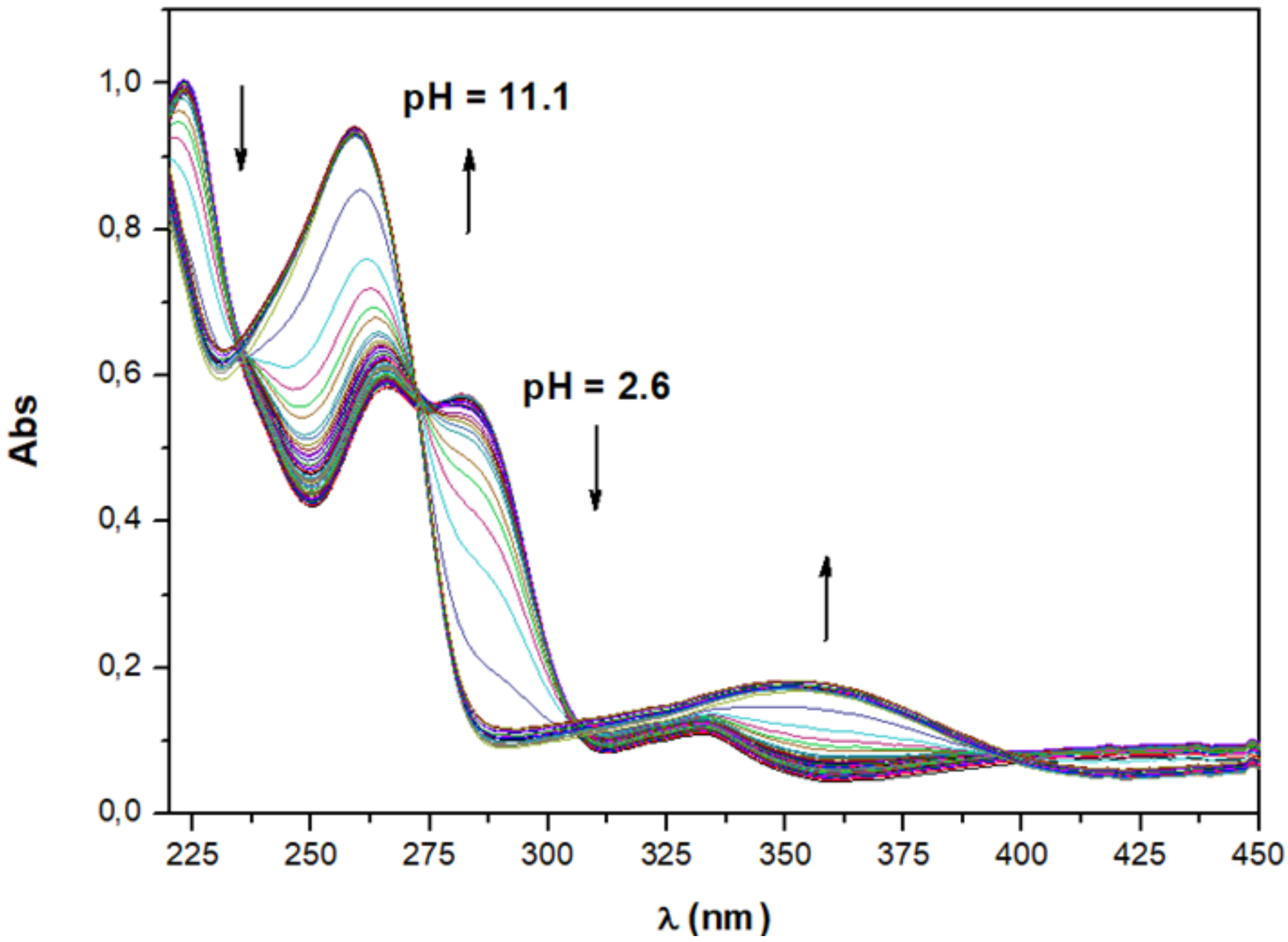

Figure S 5. UV-Vis titration of LAPQ (4.9 × 10^{-5} M) with NaOH (0.10 M) in water at 25 °C and I = 0.10 M (NaCl). Initial pH

= 2.6.

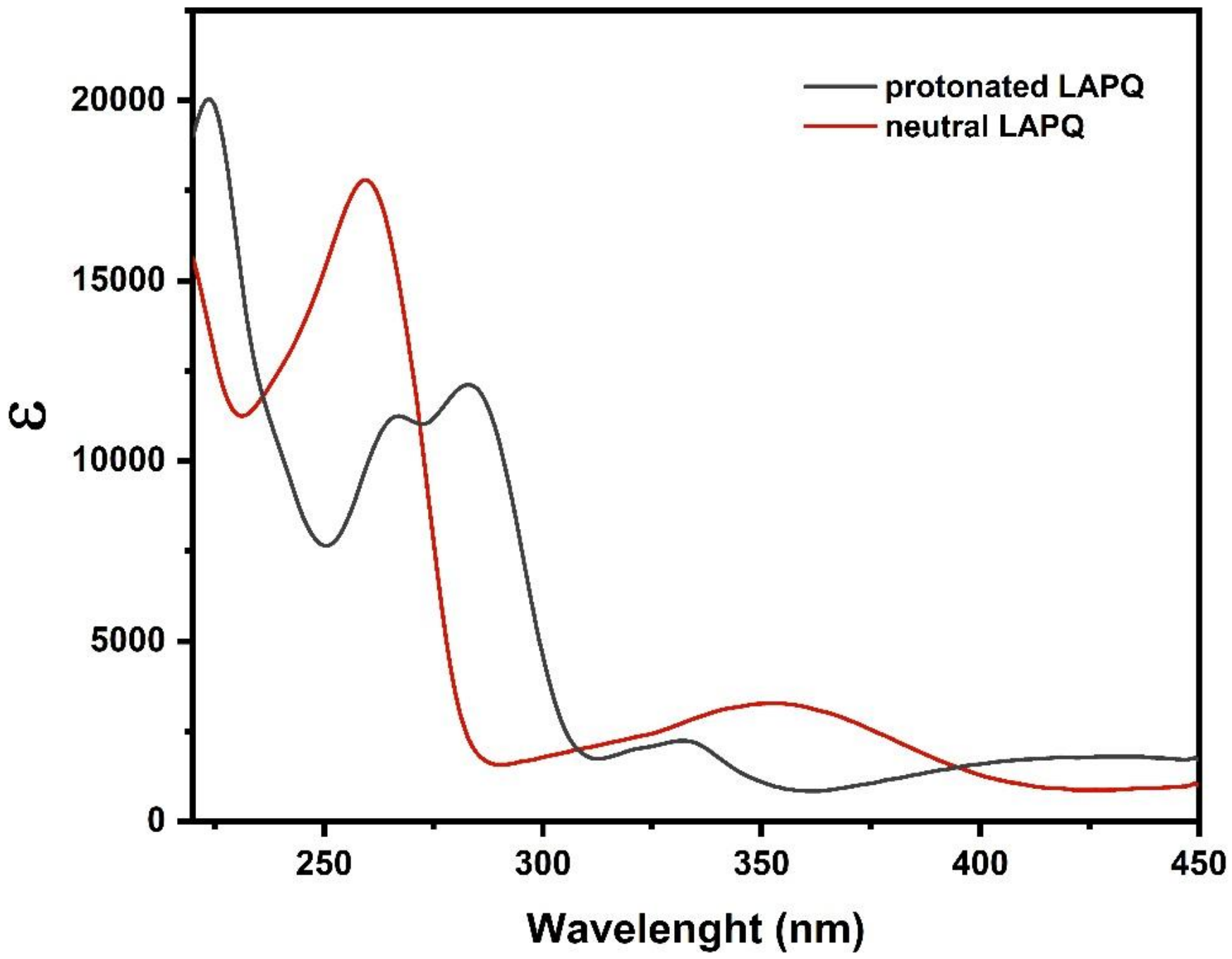

Figure S 6. Calculated molar absorptivity spectra of the protonated and neutral forms of LAPQ, obtained from the fitting of the pH-dependent UV–Vis spectrophotometric data.

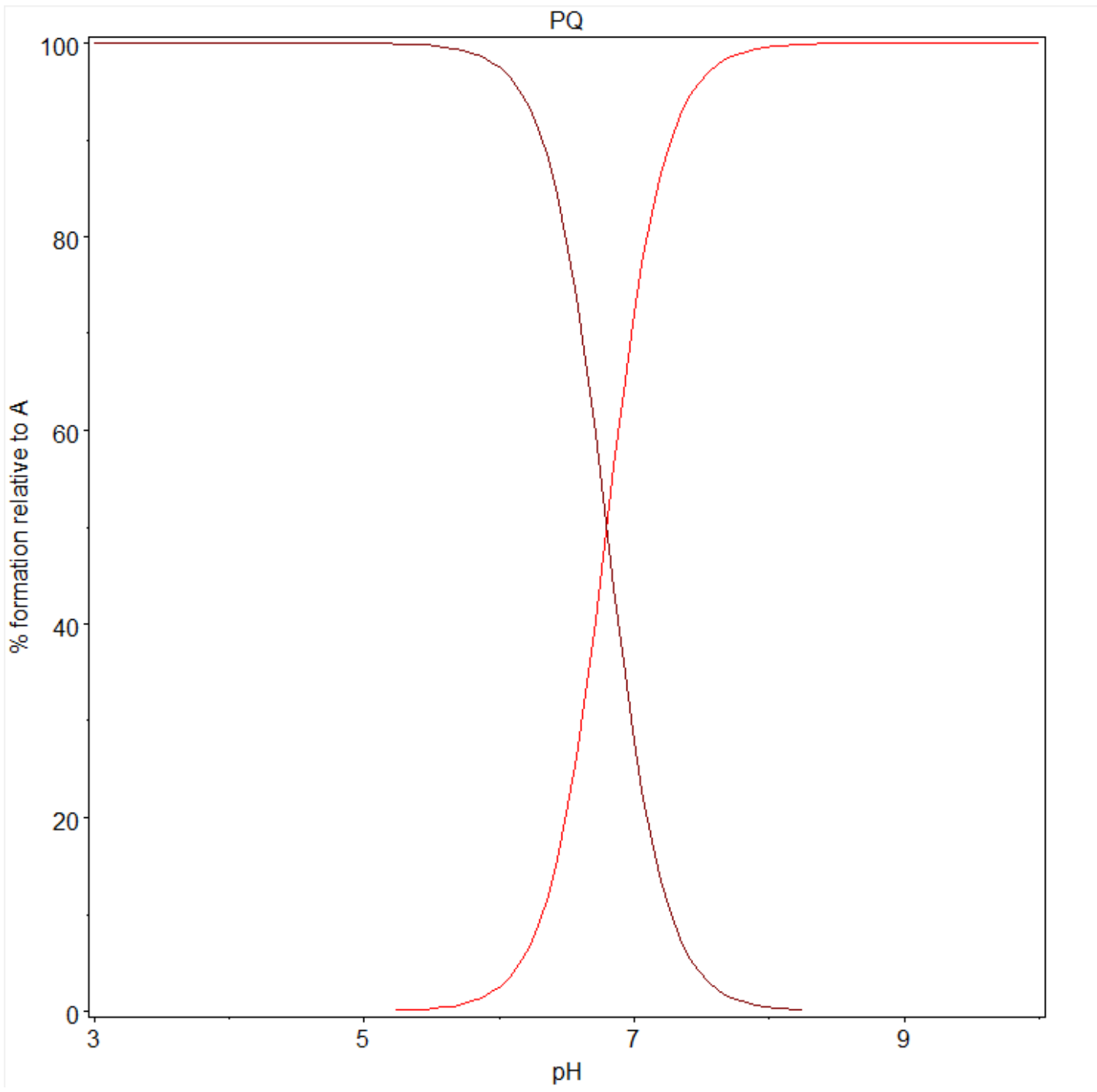

Figure S7. Species distribution diagram of PQ as a function of pH.

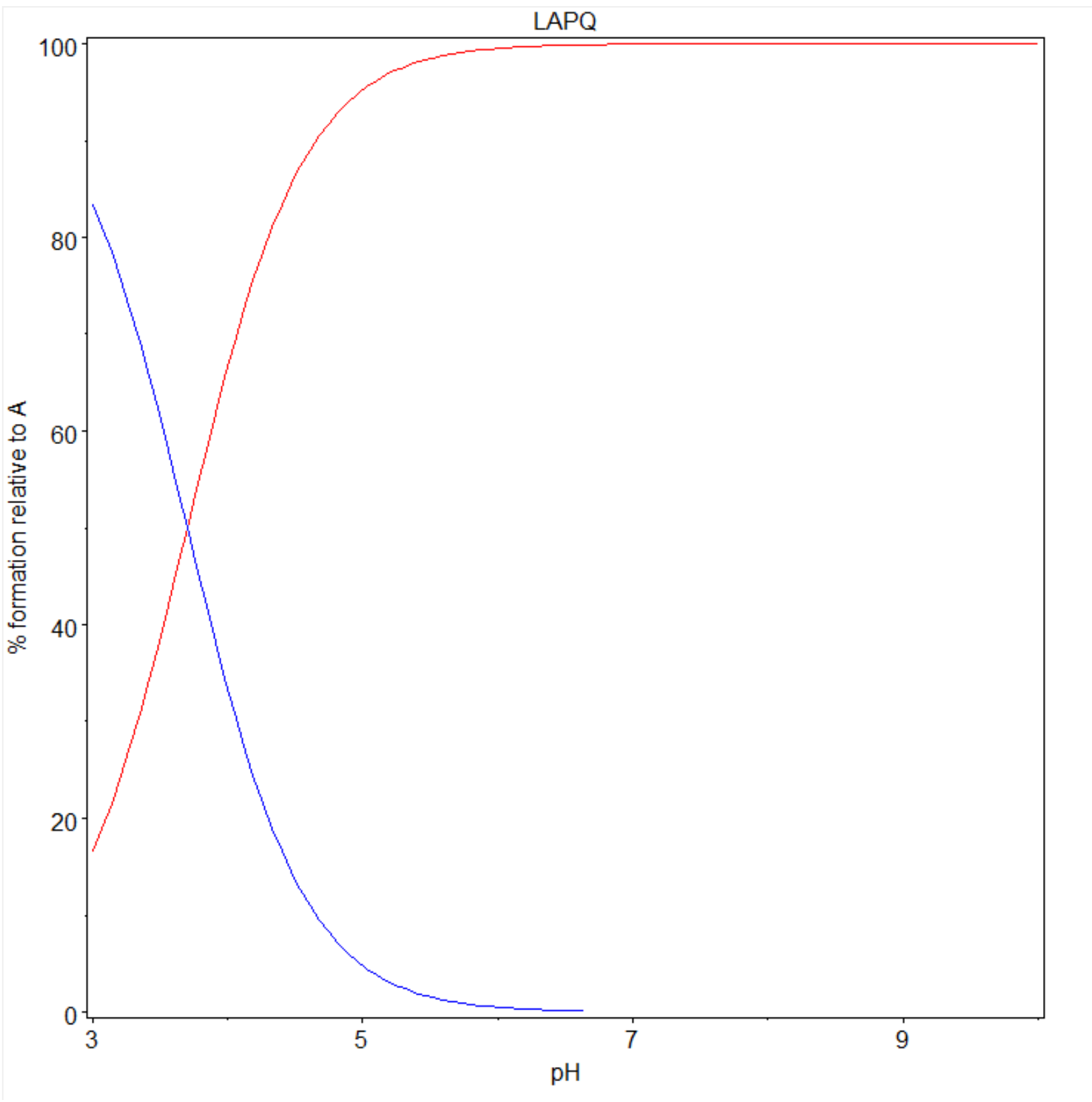

Figure S8. Species distribution diagram of LAPQ as a function of pH.

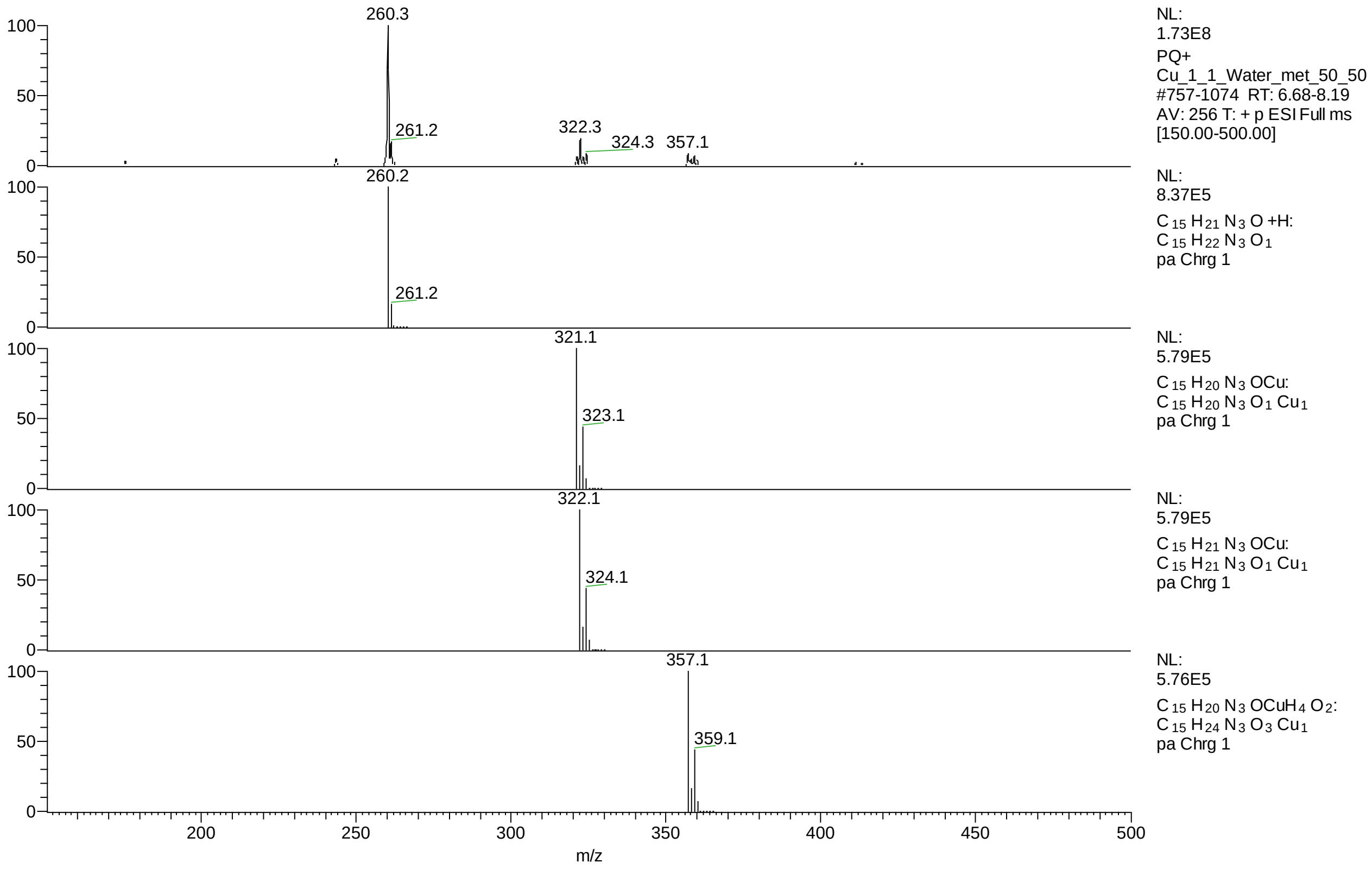

Figure S9. ESI-MS spectrum and isotopic pattern simulations of PQ/Cu^{2+} system investigated at a 1:1 metal-to-ligand ratio.

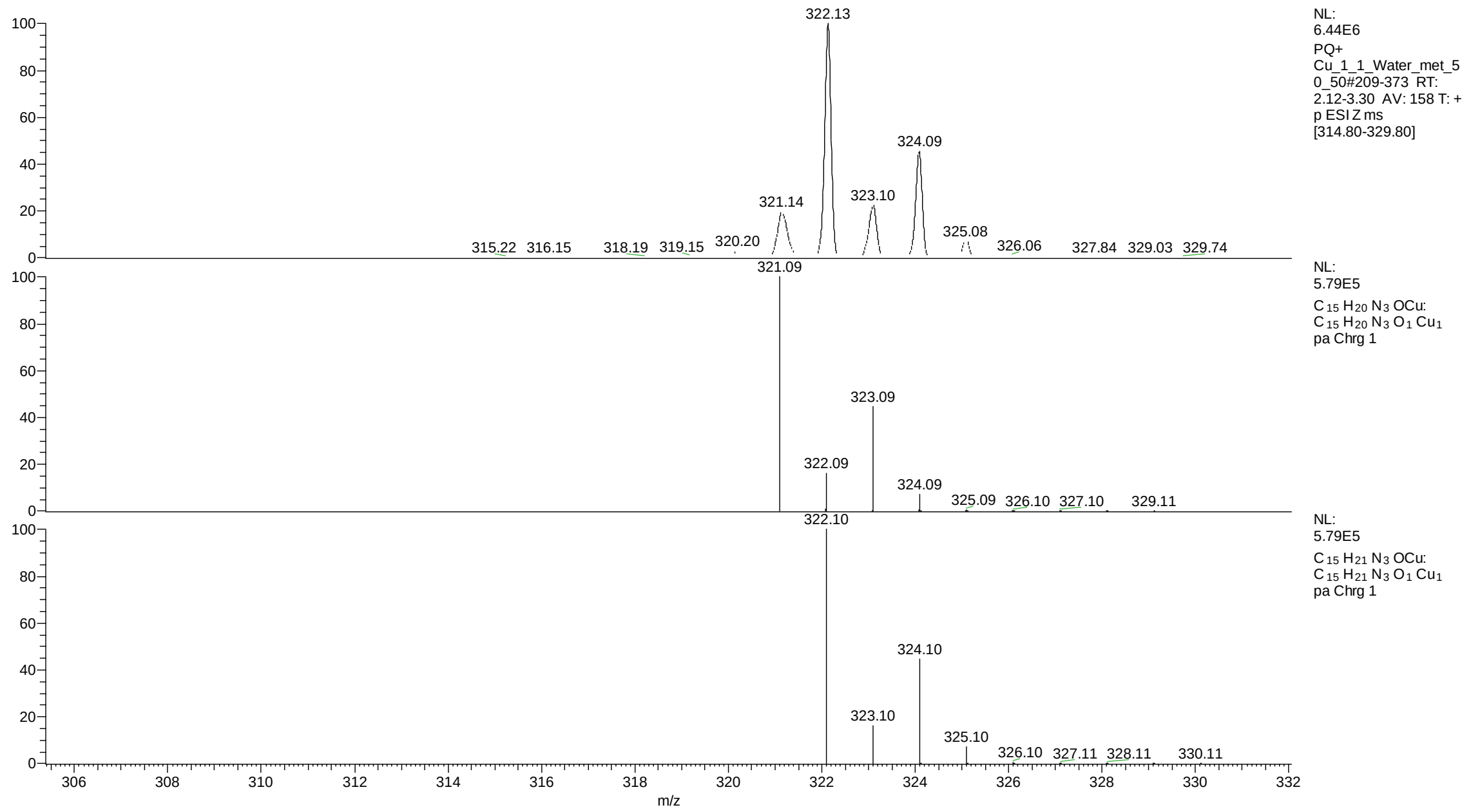

Figure S10. ESI-MS zoom scan spectrum and isotopic pattern simulations of PQ/Cu^{2+}system investigated at a 1:1 metal-to-ligand ratio.

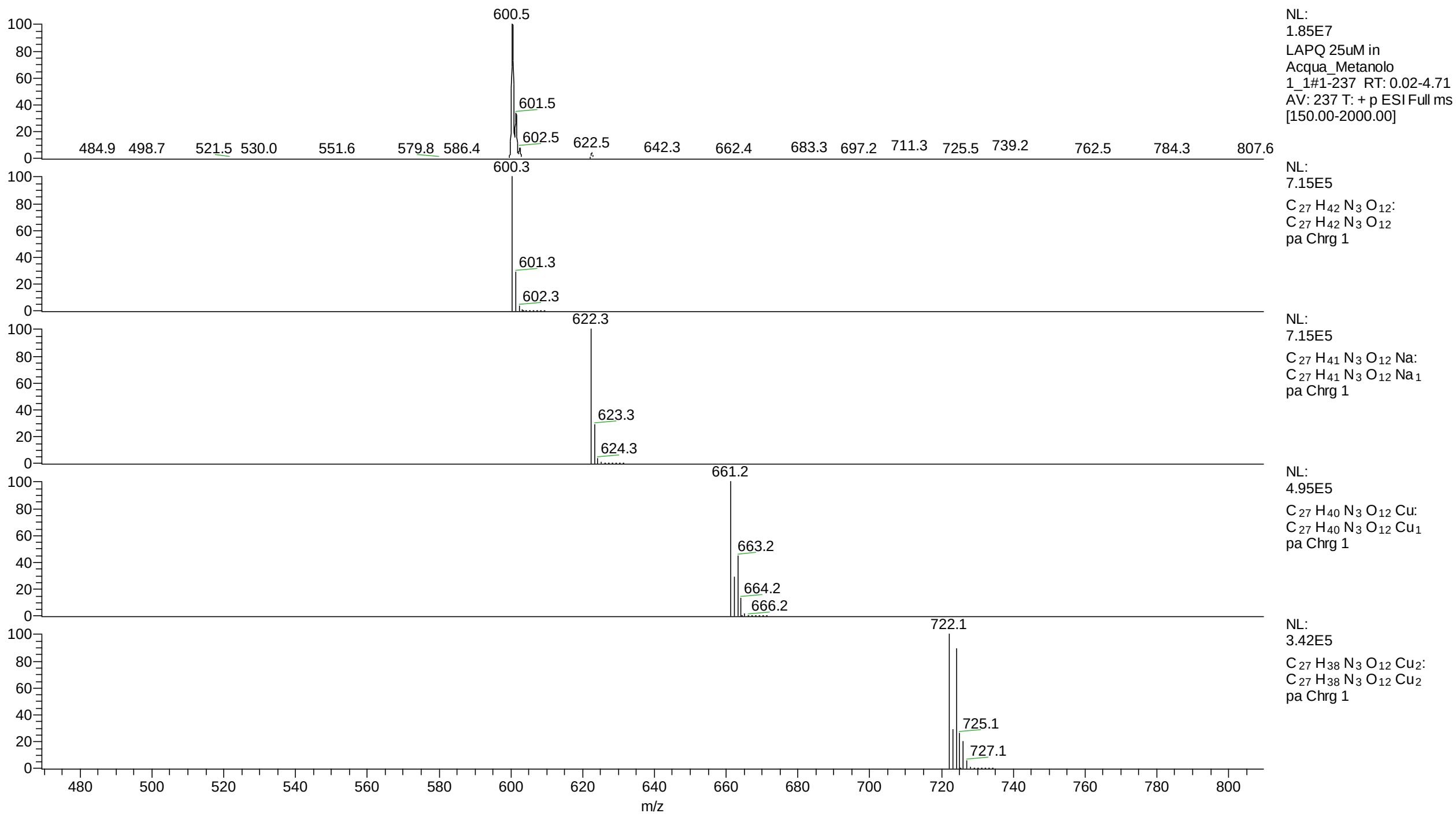

Figure S11. ESI-MS zoom scan spectrum and isotopic pattern simulations of LAPQ/Cu^{2+} system investigated at a 1:1 metal-to-ligand ratio.

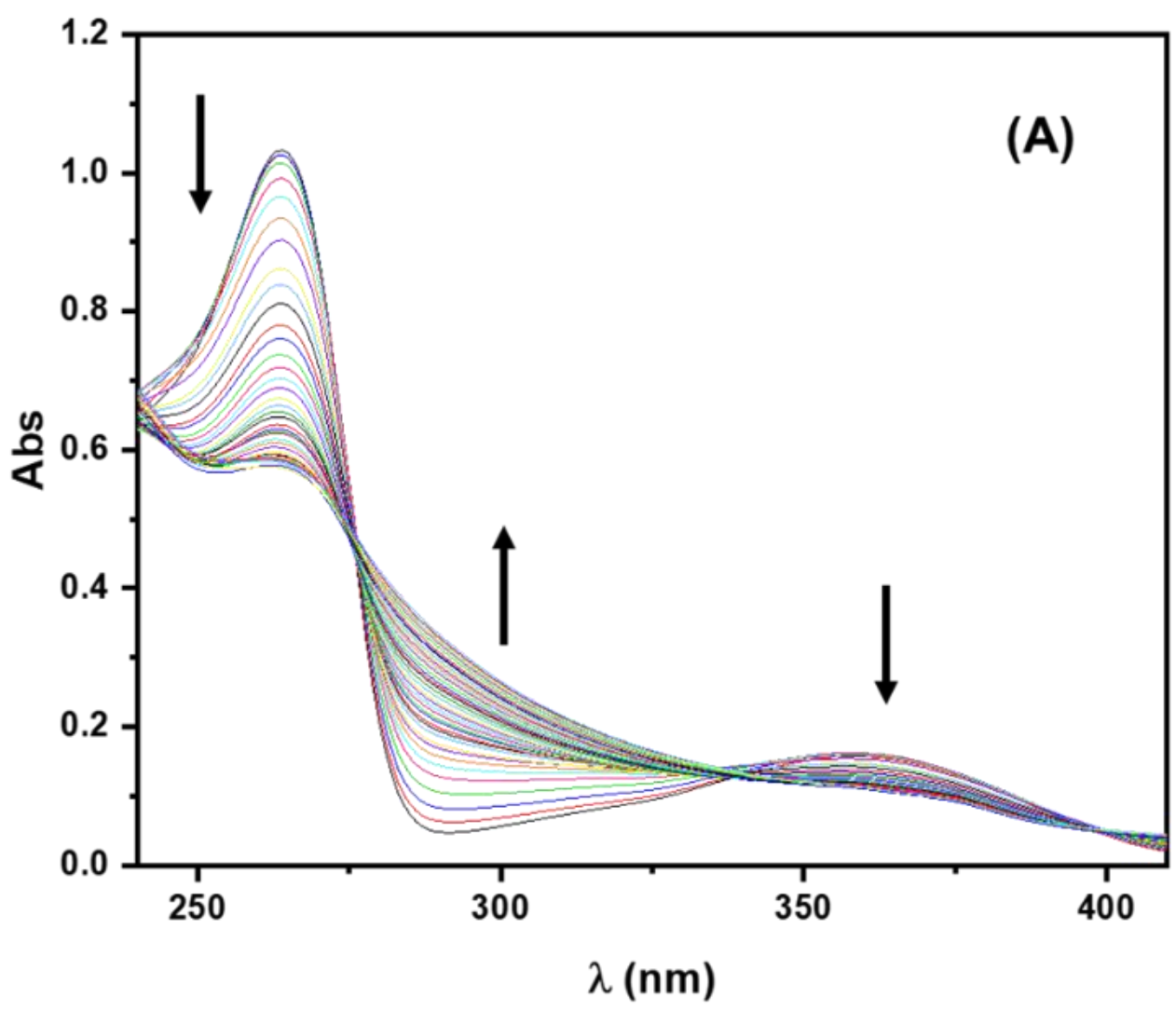

Figure S 12. UV−vis titration of PQ (5.0 x 10^{-5} M) upon addition of Cu^{2+} (0.3-3.1 mol equiv) in 0.02M MOPS/Dioxane (70:30) at pH 7.4.

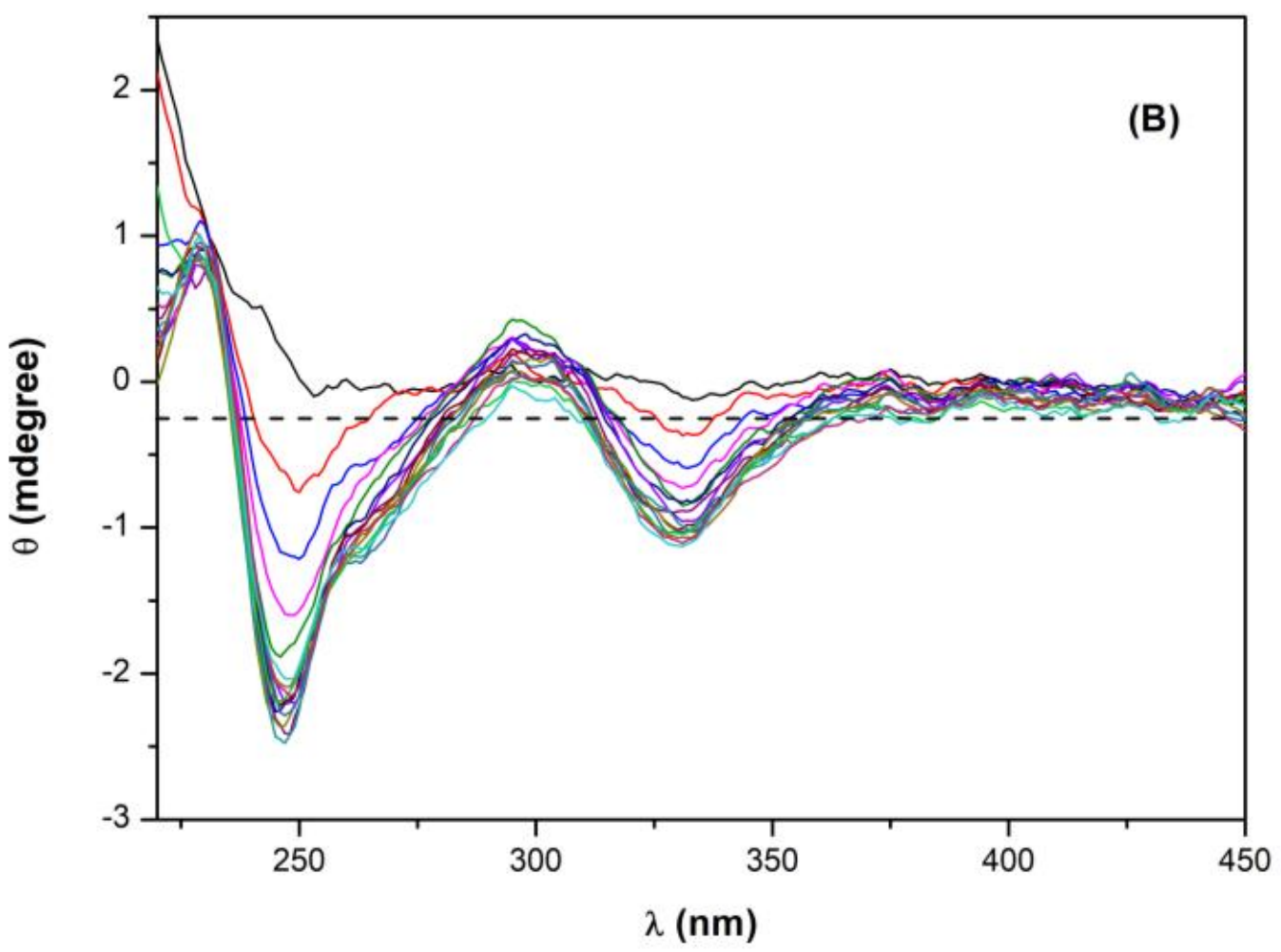

Figure S 13 CD titration of LAPQ (4.9 × 10^{-5} M) upon addition of Cu^{2+} (0.15 - 2.25 mol equiv) in 0.01 M MOPS at pH 7.4.

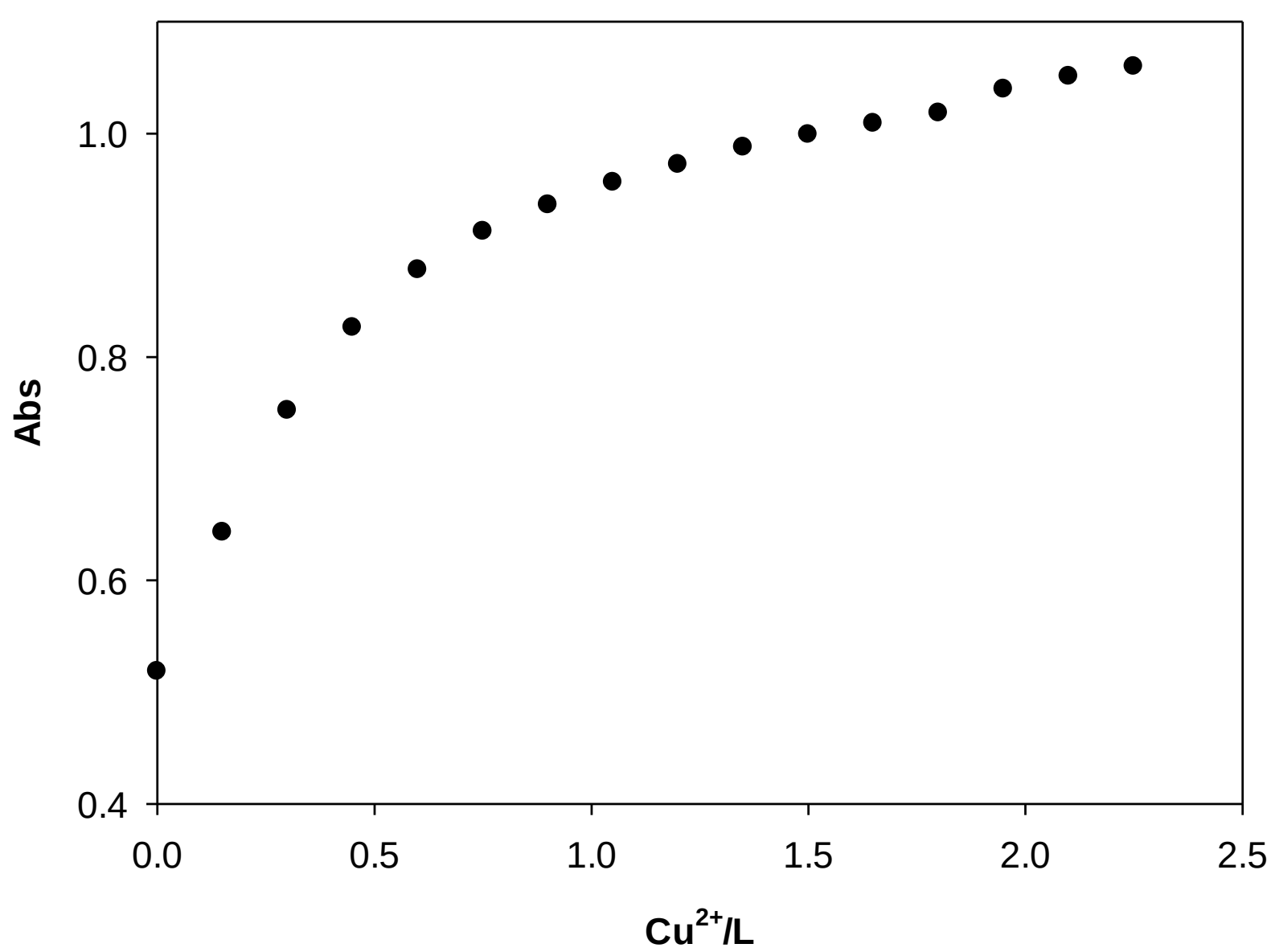

Figure S14. Collected Abs values at 264nm vs the molar ratio of [Cu]/[LAPQ].

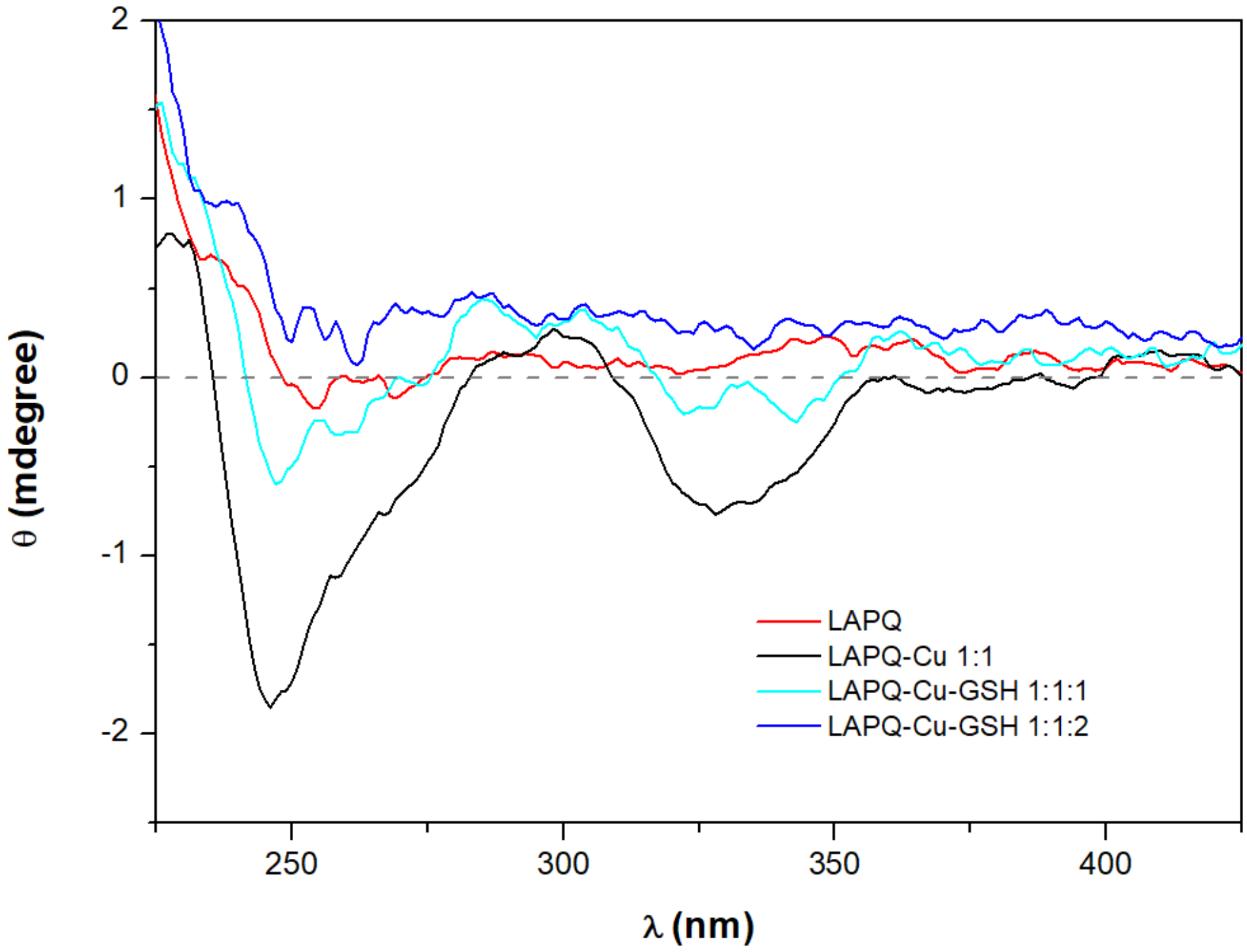

Figure S 15. CD spectra of mixtures of LAPQ alone, LAPQ/Cu^{2+} 1:1, LAPQ/Cu^{2+}/GSH 1:1:1 and LAPQ/Cu^{2+}/GSH 1:1:2 at pH 7.4 (0.01 M MOPS). $C_{LAPQ} = 4.9 \times 10^{-5}$ M.

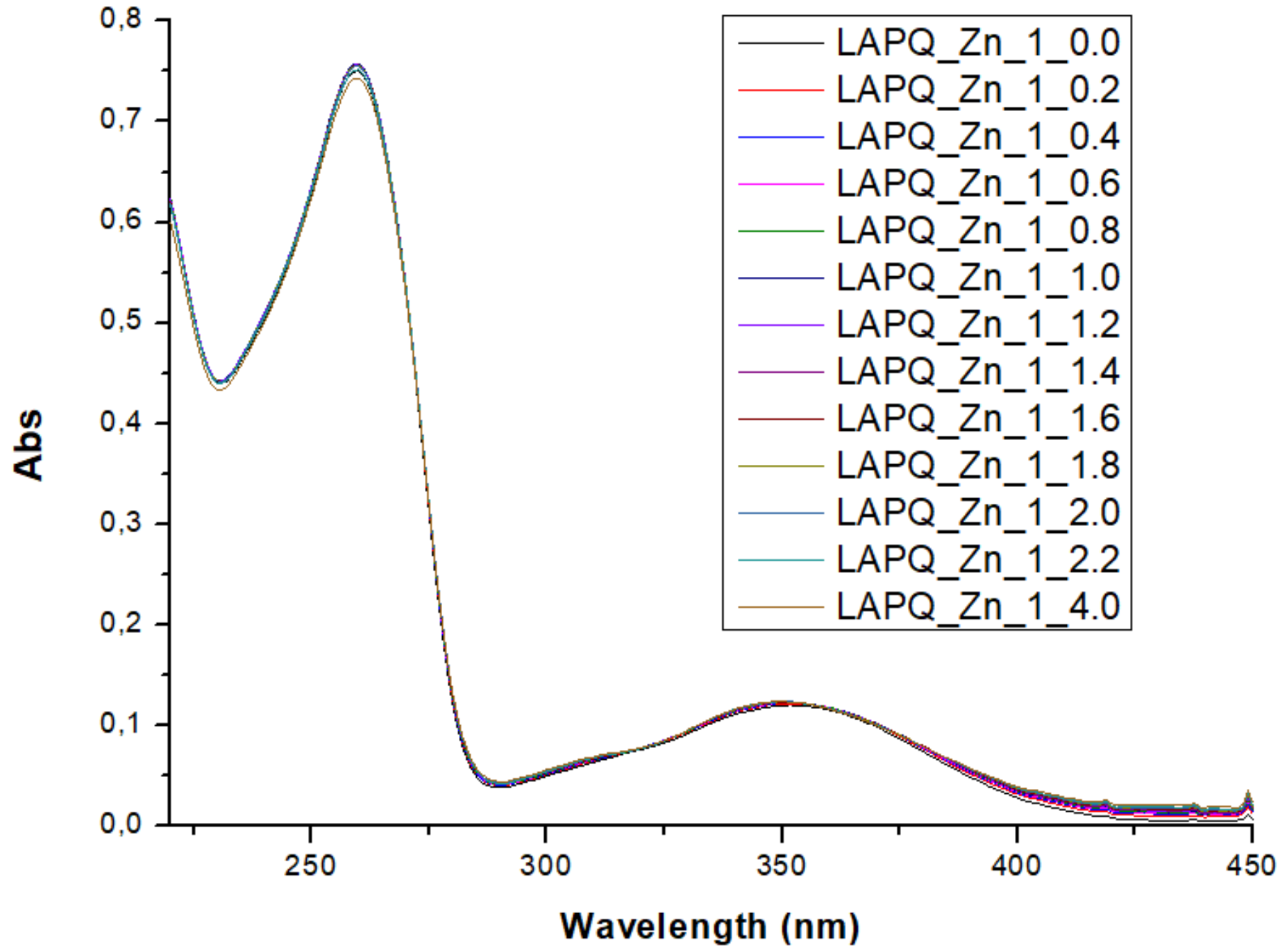

Figure S 16. UV-vis titration of LAPQ (4.9×10^{-5} M) upon addition of Zn^{2+} (0 - 4 mol equiv) in 0.01 M MOPS at pH 7.4.

Table S1. ADMET Profile of LAPQ (ADMETLab 3.0)

Physicochemical Properties

Property	*Value*	*Comment*
Molecular Weight	*599.27*	*Contain hydrogen atoms. Optimal: 100–600*
Volume	*572.533*	*Van der Waals volume*
Density	*1.047*	*Density = MW / Volume*
nHA	*15.0*	*Number of hydrogen bond acceptors*
nHD	*10.0*	*Number of hydrogen bond donors*
nRot	*16.0*	*Number of rotatable bonds*
nRing	*3.0*	*Number of rings*
MaxRing	*10.0*	*Number of atoms in the biggest ring*
nHet	*15.0*	*Number of heteroatoms*
fChar	*0.0*	*Formal charge*
nRig	*18.0*	*Number of rigid bonds*
Flexibility	*0.889*	*nRot/nRig*
Stereo Centers	*10.0*	*Number of stereocenters*
TPSA (Å²)	*243.55*	*Topological Polar Surface Area*
logS	*-2.283*	*Aqueous solubility*
logP	*0.104*	*Octanol/water partition coefficient*
logD	*0.911*	*Distribution coefficient*
Melting Point (°C)	*147.916*	*Predicted melting point*
Boiling Point (°C)	*304.928*	*Predicted boiling point*

Medicinal Chemistry

Property	*Value*
QED	*0.093*
GASA	*1.0*
Synth	*4.0*
Fsp3	*0.63*
MCE-18	*77.455*
NPscore	*0.695*
Lipinski Rule	*1.0*

Property	Value
Pfizer Rule	0.0
GSK Rule	1.0
Golden Triangle	1.0

Absorption

Property	Value
Caco-2 Permeability	-5.567
MDCK Permeability	-5.148
PAMPA	0.985
Pgp Inhibitor	0.0
Pgp Substrate	0.979
HIA	0.864
F20%	0.958
F30%	0.999
F50%	0.999

Distribution

Property	Value
PPB	69.851
VDss	-0.528
BBB	0.0
Fu	29.372
OATP1B1 Inhibitor	1.0
OATP1B3 Inhibitor	1.0
BCRP Inhibitor	0.0
MRP1 Inhibitor	0.811

Metabolism

Property	Value
CYP1A2 Inhibitor	0.0

Property	Value
CYP1A2 Substrate	0.0
CYP2C19 Inhibitor	0.001
CYP2C19 Substrate	0.999
CYP2C9 Inhibitor	0.001
CYP2C9 Substrate	0.034
CYP2D6 Inhibitor	0.005
CYP2D6 Substrate	0.001
CYP3A4 Inhibitor	0.017
CYP3A4 Substrate	0.784
CYP2B6 Inhibitor	0.0
CYP2B6 Substrate	0.0
CYP2C8 Inhibitor	0.997
HLM Stability	0.0

Excretion

Property	Value
CLplasma	1.351
T1/2	1.845

Toxicity

Property	Value
hERG Blockers	0.015
hERG Blockers (10 μM)	0.053
DILI	0.848
AMES Mutagenicity	0.924
Rat Oral Acute Toxicity	0.072
FDAMDD	0.027
Skin Sensitization	0.704
Carcinogenicity	0.061

Property	***Value***
Eye Corrosion	***0.0***
Eye Irritation	***0.0***
Respiratory Toxicity	***0.012***
Human Hepatotoxicity	***0.690***
Drug-induced Nephrotoxicity	***0.928***
Ototoxicity	***1.000***
Hematotoxicity	***0.425***
Genotoxicity	***0.990***
RPMI-8226 Immunotoxicity	***0.142***
A549 Cytotoxicity	***0.301***
HEK293 Cytotoxicity	***0.774***
Drug-induced Neurotoxicity	***0.413***

Environmental Toxicity

Property	***Value***
Bioconcentration Factors	***0.192***
IGC50	***2.757***
LC50FM	***3.522***
LC50DM	***4.674***

Tox21 Pathway

Property	***Value***
NR-AhR	***0.402***
NR-AR	***0.038***
NR-AR-LBD	***0.008***
NR-Aromatase	***0.005***
NR-ER	***0.333***
NR-ER-LBD	***0.003***
NR-PPAR-gamma	***0.0***

Property	Value
SR-ARE	0.395
SR-ATAD5	0.008
SR-HSE	0.0
SR-MMP	0.001
SR-p53	0.027

Toxicophore Rules

Property	Value
Acute Toxicity Rule	0
Genotoxic Carcinogenicity Rule	1 alert
Non-Genotoxic Carcinogenicity Rule	0
Skin Sensitization Rule	3 alerts
Aquatic Toxicity Rule	0
Non-Biodegradable Rule	2 alerts
SureChEMBL Rule	0
FAF-Drugs4 Rule	1 alert
